# Lactate‐H3K18la‐RPL11 Axis Drives Gemcitabine Resistance in Pancreatic Cancer by Integrating Protein Synthesis and DNA Damage Repair

**DOI:** 10.1002/advs.78160

**Published:** 2026-10-08

**Authors:** Lijun Zhao, Jianzhong Cao, Wenyue Liu, Xuyun Li, Haolin Zhang, Xiaofang Zhang, Shiyang Shi, Huilong Yin, Junjie Li, Angang Yang, Rui Zhang

**Affiliations:** ^1^ Key Laboratory of Holistic Integrative Management of Gastrointestinal Cancers and Department of Immunology Fourth Military Medical University Xi'an Shaanxi China; ^2^ Henan Key Laboratory of Immunology and Targeted Therapy Molecular Immunology and Immunotherapy Laboratory School of Medical Technology Xinxiang Medical University Xinxiang Henan China; ^3^ Department of Pancreatobiliary Surgery State Key Laboratory of Oncology in South China Guangdong Provincial Clinical Research Center for Cancer Sun Yat‐sen University Cancer Center Guangzhou China; ^4^ Department of Emergency Xijing Hospital Fourth Military Medical University Xi'an China

**Keywords:** DNA damage repair, gemcitabine resistance, histone lactylation, protein synthesis, RPL11

## Abstract

Acquired resistance to gemcitabine (GEM) remains a major barrier to effective treatment of pancreatic ductal adenocarcinoma (PDAC). Here we identify histone H3K18 lactylation (H3K18la), induced by GEM‐driven lactate accumulation, as a key epigenetic mechanism of chemoresistance. H3K18la functions as a transcriptional activator of ribosomal protein L11(RPL11) Elevated RPL11 establishes a dual, compartmentalized pro‐survival program: in the cytoplasm, RPL11 binds and stabilizes eukaryotic translation elongation factor 1 alpha 1 (EEF1A1) to enhance global protein synthesis; in the nucleus, RPL11 serves as a noncanonical transcriptional coactivator of (ADP‐ribose) polymerase 14 (PARP14), augmenting DNA damage repair and attenuating GEM cytotoxicity. Pharmacologic or genetic inhibition of lactate production or RPL11 expression synergizes with GEM to overcome resistance and suppress PDAC progression in xenograft models. These findings define a lactate–H3K18la–RPL11axis that couples metabolic and epigenetic remodeling to translational control and genome maintenance, revealing actionable vulnerabilities in PDAC chemoresistance.

## Graphical abstract

A lactate‐H3K18la epigenetic axis upregulates RPL11 to drive gemcitabine resistance in pancreatic cancer. RPL11 orchestrates chemoresistance through a dual, compartmentalized role: enhancing cytoplasmic protein synthesis via EEF1A1 stabilization and promoting nuclear DNA damage repair by co‐activating PARP14, thus ensuring robust cell survival.

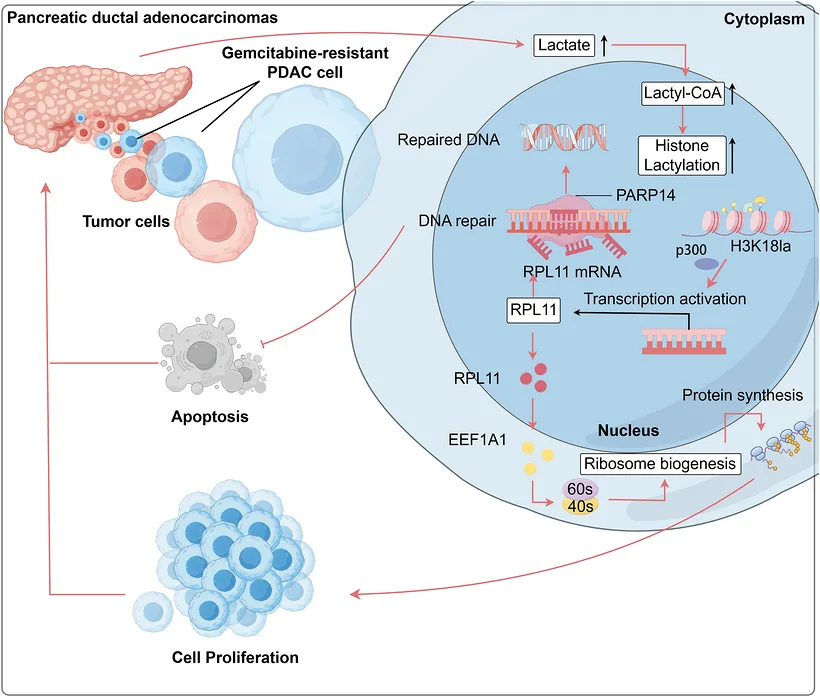

## 1 Introduction

Pancreatic ductal adenocarcinoma (PDAC) remains one of the most lethal solid malignancies, with a five‐year survival rate of less than 10% [1]. Although gemcitabine (GEM)‐based chemotherapy is a cornerstone of first‐line treatment, the rapid emergence of intrinsic or acquired resistance presents a fundamental barrier to its clinical efficacy, ultimately leading to therapeutic failure [2]. While previous studies have elucidated several resistance mechanisms, including altered drug transport and metabolic bypass pathways [2], a comprehensive understanding of how PDAC cells sense and adapt to the complex tumor microenvironment (TME) via epigenetic reprogramming to drive chemoresistance remains largely incomplete.

Metabolic reprogramming, particularly the Warburg effect characterized by high‐throughput lactate production, is a core feature of PDAC malignancy [3]. Owing to its exceptionally dense desmoplastic stroma, PDAC tumors exhibit impaired metabolite clearance, leading to local lactate concentrations as high as 10–40 mm [4]. Recent evidence has redefined lactate from a metabolic waste product to a critical signaling molecule deeply involved in tumor progression [5]. A novel post‐translational modification, histone lactylation, directly links cellular metabolism to the epigenetic regulatory network [6]. Histone lactylation (e.g., H3K18la) has been implicated in tumor immunosuppression and chemoresistance in some cancers [7, 8, 9]. For example, histone lactylation increases colorectal cancer resistance to bevacizumab by enhancing the expression of the autophagy‐promoting protein RUBCNL [10]. Its role in orchestrating specific gene expression programs to drive acquired GEM resistance in PDAC remains unknown.

To address this question, we integrated multi‐omic screening (including immunoprecipitation‐mass spectrometry (IP‐MS), RNA sequencing (RNA‐seq), and chromatin immunoprecipitation sequencing (ChIP‐seq)) with clinical cohort analysis. Our unbiased approach unexpectedly identified RPL11 as the central hub protein directly regulated by H3K18la. Aberrant enhancement of ribosome biogenesis is a critical strategy for cancer cells to meet their high anabolic demands and cope with chemotherapeutic stress [11]. Although most ribosomal proteins (RPs) function as structural scaffolds, RPL11 is distinguished by its prominent extra‐ribosomal functions, acting not only as a core ribosomal component but also as a key “stress sensor” that monitors the cellular microenvironment [12, 13, 14]. In p53 wild‐type cells, ribosomal stress prompts free RPL11 to bind and inhibit Murine Double Minute 2 (MDM2), thereby activating p53‐mediated tumor suppression [15, 16]. However, given that over 75% of PDAC cases harbor loss‐of‐function mutations in *TP53* [17], we hypothesized that RPL11 could be hijacked and reprogrammed to confer a survival advantage under therapeutic pressure. Despite its prognostic significance in various cancers [18], the precise role of RPL11 in mediating metabolically‐driven chemoresistance in PDAC has not been elucidated.

Here, we unveil a novel lactate‐H3K18la‐driven signaling axis that plays a pivotal role in mediating GEM resistance in PDAC. We demonstrate that lactate accumulation in resistant cells recruits EP300 to the RPL11 promoter, catalyzing H3K18la and inducing RPL11 overexpression. We further reveal that RPL11 executes a unique, bimodal pro‐survival program through functional compartmentalization: in the cytoplasm, RPL11 post‐translationally stabilizes the elongation factor EEF1A1 to enhance global protein synthesis; in the nucleus, it acts as a transcriptional co‐activator to directly upregulate PARP14, thereby augmenting DNA damage repair capacity. Finally, we show that combined pharmacological inhibition of this axis effectively re‐sensitizes resistant tumors to GEM. These findings not only provide a new paradigm for understanding how metabolic reprogramming drives chemoresistance but also offer a solid rationale for developing novel combination therapies targeting the metabolism‐epigenetics‐signal reprogramming axis in PDAC.

## 2 Results

### 2.1 Elevated H3K18la Is a Key Molecular Signature Driving Acquired Gemcitabine Resistance in PDAC

To investigate the clinical relevance of histone lactylation in chemoresistance of PDAC, we first analyzed a cohort of patient‐derived tumor tissue samples. This cohort comprised 10 samples from patients sensitive to GEM treatment and 10 samples from patients exhibiting resistance. Immunohistochemistry (IHC) staining revealed a significant enrichment of histone H3 lysine 18 lactylation (H3K18la) levels in resistant tumors compared to sensitive ones (Figure 1A,B). To directly corroborate the initiating role of lactate accumulation in the resistant phenotype at the clinical level, we further performed a quantitative assessment of lactate concentration in the tumor tissues of the aforementioned cohort. Analysis showed that the baseline lactate concentration within GEM‐resistant (GEM‐R) tumor tissues was significantly higher than that in the sensitive group (Figure 1C), confirming that a high‐lactate microenvironment is directly correlated with the clinical resistant phenotype. More importantly, intratumoral lactate concentration exhibited a significant positive correlation with the H3K18la IHC score (Figure 1D), indicating that intratumoral lactate accumulation serves as the upstream metabolic basis driving the aberrant enrichment of H3K18la.

**FIGURE 1:**
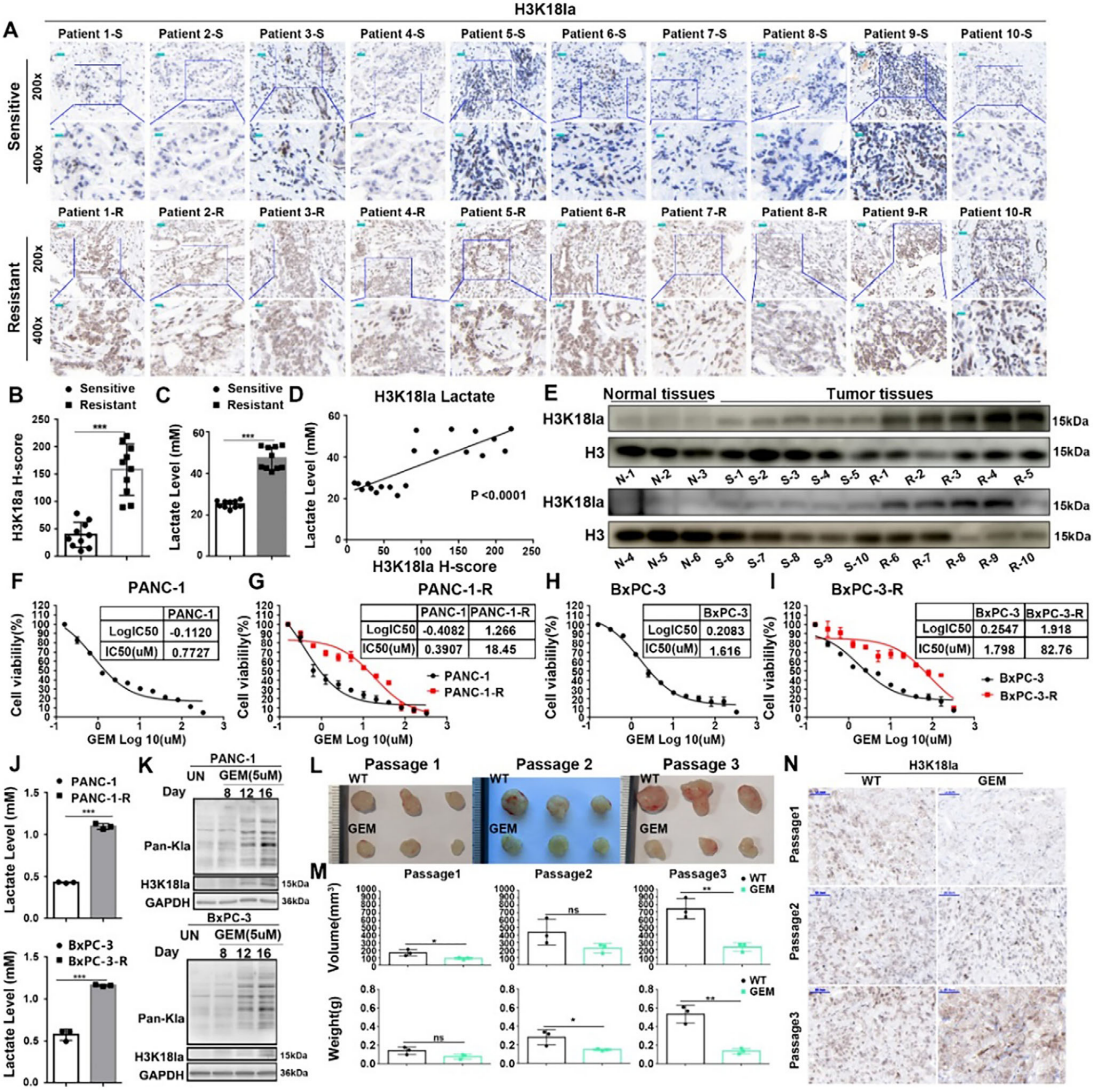
Aberrant enrichment of histone H3K18 lactylation (H3K18la) is closely associated with acquired gemcitabine (GEM) resistance and lactate accumulation in PDAC. (A) Representative immunohistochemistry (IHC) images of H3K18la levels in tumor tissues from 10 pairs of clinically matched GEM‐sensitive and ‐resistant PDAC patients. Scale bars: 50 µm (top, 200×) and 20 µm (bottom, 400×). (B) Quantitative analysis of H3K18la expression intensity (H‐score) in sensitive and resistant PDAC tissues (*n* = 10/group). (C) Quantitative measurement of absolute lactate concentrations in tumor tissue homogenates from sensitive and resistant groups (*n* = 10/group). (D) Pearson correlation analysis between lactate concentrations and H3K18la H‐scores in clinical PDAC tumor tissues (*n* = 20). (E) Western blot analysis of H3K18la expression in normal pancreatic tissues and matched GEM‐sensitive (S‐1 to S‐10) and ‐resistant (R‐1 to R‐10) PDAC samples. Total histone H3 served as an internal control. (F–I) Dose‐response cell viability curves and IC50 values of parental PDAC cell lines (PANC‐1, BxPC‐3) and acquired GEM‐resistant cell lines (PANC‐1‐R, BxPC‐3‐R) under various GEM concentrations. (J) Quantitative comparison of basal intracellular lactate concentrations between parental and resistant PDAC cell lines (*n* = 3). (K) Western blot analysis of dynamic changes in Pan‐Kla and H3K18la levels in parental cells continuously treated with 5 µm GEM for indicated times (8, 12, 16 days; UN represents untreated control). GAPDH served as an internal control. (L–N) Validation of the acquired resistance model established via in vivo cyclic GEM screening across three consecutive passages (Passage 1–3) using BxPC‐3 xenografts. (L) Gross images of representative endpoint tumors (WT: control group, GEM: treatment group). (M) Quantification of endpoint tumor weights and volumes across passages (*n* = 3/group). (N) Representative IHC images of H3K18la in xenograft tissues across the three passages. Data are presented as mean ± standard deviation (SD). Comparisons between two groups were evaluated by two‐tailed Student's t‐test; correlations were assessed by Pearson correlation analysis. ^*^p < 0.05, ^**^p < 0.01, ^***^p < 0.001, ^****^p < 0.0001.

Furthermore, to elucidate the baseline levels of histone lactylation in PDAC and its dynamic alteration characteristics during the acquisition of resistance, we included normal pancreatic tissues as controls. Western blot analysis demonstrated that, compared to normal tissues, both pan‐lysine lactylation (Pan‐Kla) and H3K18la already exhibited a significant baseline elevation in GEM‐sensitive PDAC tissues, which perfectly aligns with the characteristics of lactylation modifications driven by the highly glycolytic nature of PDAC [19]. However, the signal intensity of H3K18la was further upregulated in GEM‐R tissues (Figure 1E and Figure S1A). Preliminary screening of multiple potential lactylation sites on histone H3 also confirmed that H3K18la is one of the most stably induced modifications among them (Figure S1A). Collectively, these clinical data establish a strong positive correlation between elevated H3K18la levels and acquired GEM resistance in PDAC.

To systematically validate the aforementioned clinical observations in vitro, we successfully established PDAC cell lines with acquired GEM resistance characteristics (PANC‐1‐R, BxPC‐3‐R, and Panc02‐R) through long‐term, dose‐escalating GEM treatment. Cell viability assays and half‐maximal inhibitory concentration (IC50) analyses confirmed a drastic reduction in GEM sensitivity in GEM‐R cells compared to their parental counterparts (Figure 1F–I and Figure S1B,C). In exploring the metabolic drivers of this resistant phenotype, we found that the basal intracellular lactate concentrations in all GEM‐R cells were significantly higher than those in their respective parental cells (Figure 1J and Figure S1D), suggesting that intracellular lactate accumulation is closely linked to the evolution of acquired resistance. Moreover, we observed that GEM treatment itself could induce a time‐dependent upregulation of Pan‐Kla and H3K18la levels in parental PDAC cells (Figure 1K and Figure S1E), indicating that GEM‐induced chemotherapeutic stress directly triggers epigenetic remodeling characterized by histone lactylation.

To validate this mechanism in a more physiologically relevant in vivo setting, we established a mouse subcutaneous xenograft model using a human PDAC cell line (BxPC‐3) combined with high‐concentration Matrigel (rich in extracellular matrix proteins to simulate the dense desmoplastic microenvironmental barrier of PDAC). Through three consecutive cycles of in vivo GEM treatment, we successfully simulated the in vivo evolutionary process of acquired resistance (Figure 1L). The results showed that in the first generation (Passage 1) model, GEM treatment significantly inhibited tumor growth; however, as in vivo pharmacological selection pressure persisted and the number of passages increased, the sensitivity of tumors to GEM progressively decreased. By the third generation (Passage 3), tumors in the GEM‐treated group still maintained vigorous growth kinetics (Figure 1L,M), indicating the successful establishment of the in vivo acquired resistance phenotype. Critically, IHC analysis of tumor tissues from each passage revealed a progressive upregulation in the abundance of H3K18la along the in vivo resistance evolutionary trajectory, peaking in the Passage 3 tumors where the resistant phenotype was most pronounced (Figure 1N).

In summary, multidimensional evidence from clinical cohorts, in vitro GEM‐R cell models, and in vivo multi‐generational resistance evolution models demonstrate that the aberrant enrichment of H3K18la, driven by lactatic metabolic reprogramming, is not merely a direct product of PDAC in response to chemotherapeutic stress, but a core molecular event that drives and characterizes acquired GEM resistance in PDAC.

### 2.2 Lactate Is the Core Effector Molecule Driving H3K18la and Sustaining the GEM‐Resistant Phenotype

Given the close association between H3K18la and GEM resistance, we further investigated whether this modification acts as a functional driver. To simulate the authentic lactate‐rich TME of PDAC, which is caused by high glycolysis and a dense desmoplastic stroma, we first exogenously supplemented three parental PDAC cell lines (BxPC‐3, PANC‐1, and Panc02) with a gradient concentration of sodium L‐lactate that matches pathophysiological characteristics. The results demonstrated that even at a concentration of 10 mm, which perfectly aligns with the baseline level within authentic PDAC tumors, intracellular total Pan‐Kla and H3K18la levels were already significantly upregulated in a prominent dose‐dependent manner (Figure 2A,K and Figure S1F). Functionally, pretreatment with pathological concentrations of lactate significantly increased the IC50 of GEM in parental cells, directly inducing a chemoresistant phenotype (Figure 2B,L and Figure S1G). Furthermore, exogenous lactate loading not only significantly accelerated the cellular proliferation rate (Figure 2E,O and Figure S1N) but also exerted a pronounced protective effect on the cells, effectively suppressing baseline cellular apoptosis (Figure 2F,G,P,Q and Figure S1O).

**FIGURE 2:**
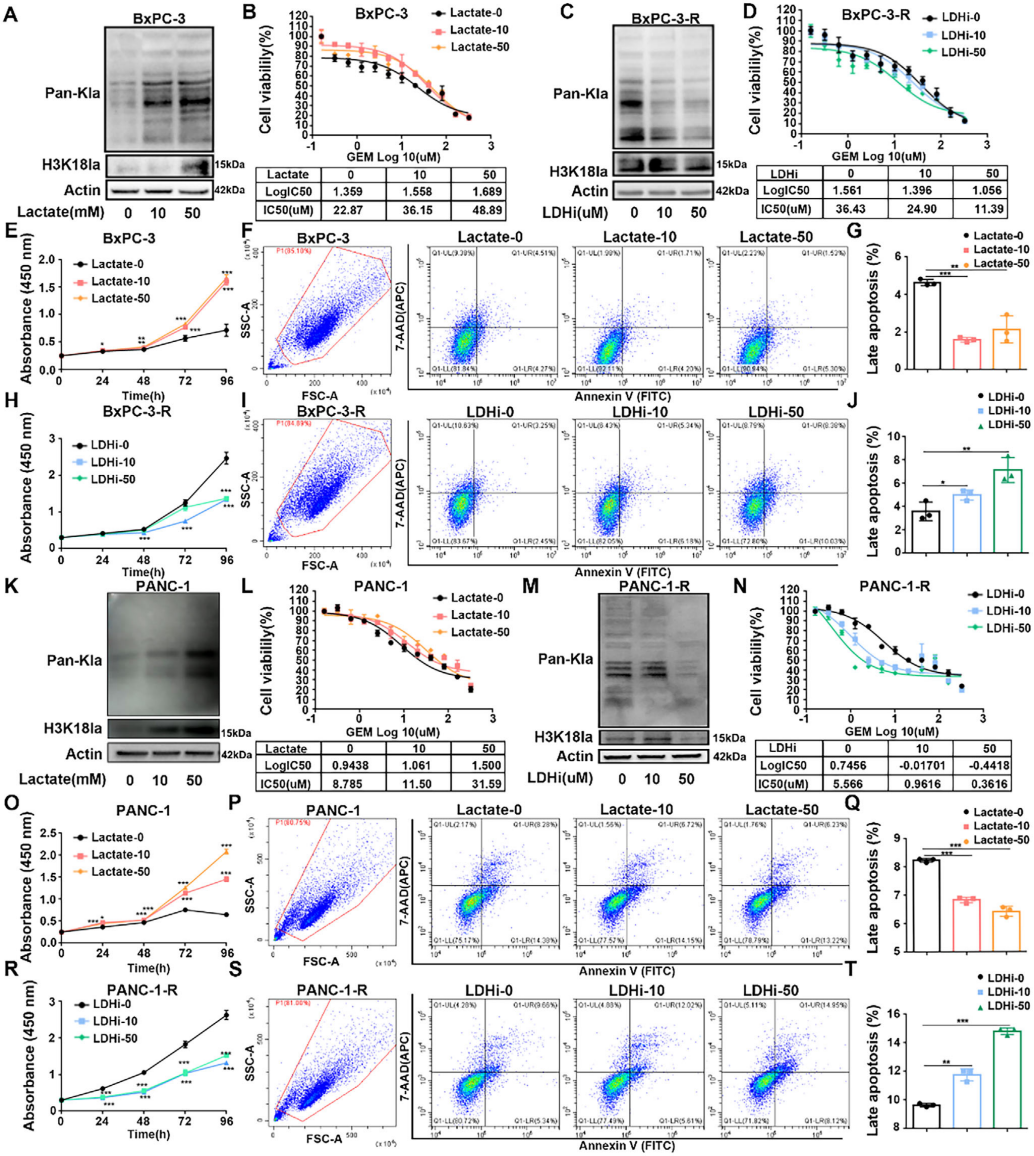
Lactate mediates gemcitabine resistance and malignant phenotypes in PDAC cells via H3K18la regulation. (A,B,E–G) Exogenous lactate induces H3K18la and drives chemoresistance and proliferation. (A) Western blot analysis shows that sodium L‐lactate (0‐50 mm, 48 h) dose‐dependently increases Pan‐Kla and H3K18la levels in BxPC‐3 cells. (B) IC50 values of GEM in BxPC‐3 cells following lactate pretreatment. (E) CCK‐8 assay reveals that lactate promotes BxPC‐3 cell proliferation. (F,G) Flow cytometry analysis of apoptosis (Annexin V/7‐AAD) and quantification of late apoptosis rates. (C,D,H–J) Inhibition of endogenous lactate production reverses resistance and suppresses malignant phenotypes. (C) The LDHA inhibitor (LDHi, 0–50 µm, 48 h) dose‐dependently reduces basal Pan‐Kla and H3K18la levels in BxPC‐3‐R cells. (D) LDHi resensitizes BxPC‐3 cells to GEM, decreasing the IC50 values. (H) LDHi inhibits the proliferation of BxPC‐3‐R cells. (I,J) Flow cytometry analysis demonstrates that LDHi induces significant apoptosis in BxPC‐3‐R cells; (J) shows the quantification of late apoptosis rates. (K–T) Consistent validation of lactate‐regulated phenotypes in the PANC‐1 cell line. Effects of exogenous lactate (K,L,O–Q) and LDHi treatment (M,N,R–T) on PANC‐1 cells. Data are presented as mean ± SD (*n* = 3 independent experiments). Statistical significance was evaluated by two‐tailed Student's t‐test. ^*^p < 0.05, ^**^p < 0.01, ^***^p < 0.001.

Subsequently, to clearly determine whether blocking the lactylation pathway could effectively overcome established chemoresistance, we rigorously conducted loss‐of‐function experiments using acquired GEM‐R cell lines (BxPC‐3‐R, PANC‐1‐R, and Panc02‐R) established through long‐term screening. We utilized a lactate dehydrogenase A inhibitor (LDHi) to block endogenous lactate production at the metabolic source. The results indicated that LDHi treatment effectively downregulated the aberrantly elevated baseline levels of Pan‐Kla and H3K18la in GEM‐R cells (Figure 2C,M and Figure S1H). Crucially, LDHi treatment profoundly restored the sensitivity of GEM‐R cells to GEM, manifested by a sharp and significant drop in IC50 values; this confirmed that the effect was not merely general additive cytotoxicity, but rather a genuine reversal of the resistant phenotype (Figure 2D,N and Figure S1I).

To further rule out the off‐target effects of pharmacological inhibitors and establish the causal status of lactate as the core driving factor, we constructed an LDHA stable knockdown cell line (LDHA‐KD) in Panc02‐R cells (Figure S1J). The results showed that LDHA depletion led to a significant decrease in endogenous H3K18la levels within the resistant cells and significantly reversed the resistant phenotype (Figure S1K), confirming that LDHA‐mediated lactate production is the primary source for maintaining the high H3K18la levels in resistant cells. To further delineate the unique core status of lactate relative to other metabolic intermediates, we performed rigorous metabolite rescue experiments in LDHA‐KD cells. After 48 h of treatment with exogenous sodium L‐lactate (20 mm), sodium acetate (10 mm, providing acetyl‐CoA precursors), or sodium pyruvate (10 mm), the results demonstrated that only the supplementation of exogenous lactate could significantly “rescue” and restore the H3K18la levels in LDHA‐KD cells, leading to a substantial rebound in their resistance to GEM. In contrast, although sodium acetate theoretically sustains the pool for histone acetylation modifications, it failed to induce an upregulation of H3K18la and completely failed to restore the resistant phenotype (the IC50 remained at a low baseline level). Similarly, supplementation with sodium pyruvate, the upstream substrate of LDHA, also failed to significantly restore H3K18la levels or the resistant phenotype (Figure S1L,M). Additionally, inhibiting endogenous lactate production significantly impaired the in vitro proliferation capacity of GEM‐R cells (Figure 2H,R and Figure S1P) and strongly induced cellular apoptosis (Figure 2I,J,S,T and Figure S1Q).

In summary, these data demonstrate that lactate (rather than other metabolic intermediates such as acetyl‐CoA or pyruvate) is the indispensable core effector molecule driving H3K18la modification and maintaining the GEM‐R phenotype. Lactylation modifications, represented by H3K18la, serve precisely as the crucial functional driving nodes that link metabolic reprogramming to the evolution of GEM resistance, as well as to malignant phenotypes such as pro‐proliferation and anti‐apoptosis in PDAC.

### 2.3 H3K18la Directly Activates RPL11 Transcription via EP300 Recruitment to Drive Ribosome Biogenesis

To systematically identify the downstream effector factors mediating the pro‐resistance function of H3K18la, we adopted an unbiased proteomic strategy and performed bioinformatic analysis on data obtained from H3K18la immunoprecipitation coupled with IP‐MS (Figure S2A). Protein–protein interaction (PPI) network analysis revealed that the ribosomal protein RPL11 is not merely a generic interacting node, but rather functions as a highly connected core hub protein (Figure 3A and Table S2). Concurrently, pathway enrichment analysis of transcriptome sequencing data from lactate‐induced pancreatic cancer cells (GSE290845) showed that differentially expressed genes were significantly enriched in the “ribosome biogenesis” and “translational regulation” pathways. Notably, a series of ribosome‐associated genes, represented by RPL11, exhibited a highly coordinated upregulation trend under lactate loading (Figure S2B,C). Given the pivotal role of RPL11 in ribosomal stress and extraribosomal signal transduction [18], we pinpointed it as the core candidate target regulated by H3K18la.

**FIGURE 3:**
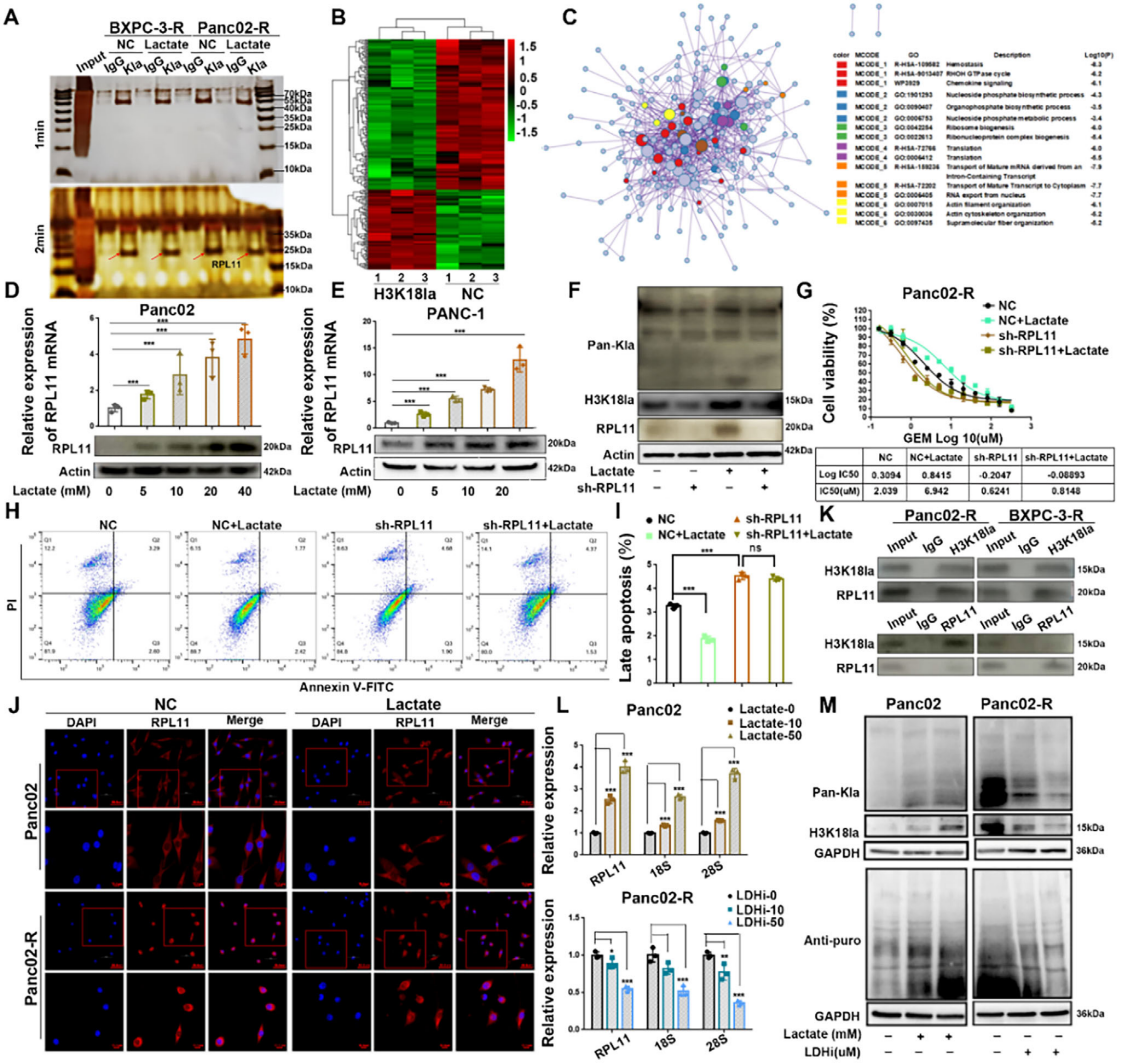
Lactate‐induced H3K18la drives RPL11 transcription via epigenetic remodeling to promote ribosome biogenesis. (A) Protein‐protein interaction (PPI) network identifies RPL11 as a core hub protein in the H3K18la interactome. (B–D) Lactate induces RPL11 upregulation and nuclear accumulation. (B) Western blot showing dose‐dependent induction of H3K18la and RPL11 proteins by exogenous lactate (0–80 mm, 48 h) in BxPC‐3 and BxPC‐3‐R cells, which is reversed by LDHi (0‐80 µm, 48 h). (C) Immunofluorescence (IF) staining reveals significant nuclear enrichment of RPL11 (red) in lactate‐treated BxPC‐3 or GEM‐resistant BxPC‐3‐R cells. Nuclei were counterstained with DAPI (blue). Scale bars: 50 µm (400×) and 10 µm (1600×). (D) Nuclear‐cytoplasmic fractionation and Western blot confirm nuclear RPL11 accumulation under lactate treatment or acquired resistance. H3 and GAPDH served as nuclear and cytoplasmic controls, respectively. (E–J) H3K18la enriches at the RPL11 promoter to mediate its transcriptional activation. (E–H) Bioinformatic analysis of public ChIP‐seq data (GSE290654). Heatmap (E) and pie chart (F) show genome‐wide H3K18la enrichment primarily at promoter regions near transcription start sites (TSS). (G) Average H3K18la signal profile across TSS regions. (H) Integrative Genomics Viewer (IGV) tracks visualizing specific H3K18la enrichment peaks at the RPL11 promoter. (I,J) ChIP‐qPCR validation shows significantly higher enrichment of H3K18la (I) and histone acetyltransferase p300 (EP300) (J) at the RPL11 promoter (P3 site) in GEM‐R BxPC‐3 cells compared to parental cells. IgG served as a negative control. (K–N) The lactate‐H3K18la‐RPL11 axis positively regulates ribosome biogenesis and global translation. (K,L) RT‐qPCR showing that exogenous lactate promotes the transcription of RPL11 and 18S/28S precursor rRNAs (K), whereas LDHi suppresses this transcription (L). (M,N) SUnSET assay demonstrating, via Western blot detection of puromycin incorporation, that lactate enhances global protein synthesis rates (M), while LDHi inhibits this process (N). Data are presented as mean ± SD (*n* = 3). Statistical significance was evaluated by two‐tailed Student's t‐test. ^***^p < 0.001.

To validate this finding under pathophysiologically relevant concentrations, we first established a strict low‐dose lactate concentration gradient in PDAC parental cells. Western blot and qPCR results demonstrated that even at a lactate concentration of 10 mm, which simulates the authentic intratumoral microenvironment, the induction of H3K18la levels and the upregulation of RPL11 expression remained highly biologically significant (Figure 3B and Figure S2D,E). To further ascertain whether RPL11 is an indispensable effector hub mediating resistance downstream of H3K18la, we constructed stable RPL11‐knockdown (sh‐RPL11) cell lines and conducted an exogenous lactate stimulation “bypass‐exclusion” assay. The results showed that although exogenous lactate stimulation successfully induced a significant elevation of H3K18la levels in shRPL11 cells (indicating an intact epigenetic response mechanism), the IC50 value for GEM remained firmly at baseline, failing to exhibit any restoration of the resistant phenotype (Figure S2F,G). Flow cytometry analysis also confirmed that while lactate stimulation significantly antagonized GEM‐induced apoptosis in control cells, this pro‐survival protective effect was entirely abolished in sh‐RPL11 cells (Figure S2H,I). These results compellingly demonstrate that RPL11 is the inevitable and irreplaceable mechanistic hub linking lactate stress to the chemoresistant phenotype.

Meanwhile, immunofluorescence (IF) and nuclear/cytoplasmic fractionation assays confirmed that either exogenous lactate treatment or acquired GEM‐R drove a significant accumulation of RPL11 within the nucleus (Figure 3C,D and Figure S2J), suggesting its role in the early stages of ribosome biogenesis or intranuclear stress signal transduction. Subsequently, to explore whether there is a physical association between H3K18la and RPL11 within the nucleus, we performed endogenous co‐immunoprecipitation (Co‐IP) analyses using nuclear extracts from GEM‐R cells. The results verified that the anti‐H3K18la antibody specifically co‐precipitated RPL11, and vice versa; this provides robust biochemical evidence for a direct or indirect physical interaction between the two within chromatin regions (Figure S2K).

Given that H3K18la is a classical transcription‐activating epigenetic modification, we further investigated whether it directly drives RPL11 expression at the transcriptional level. Mining of a ChIP‐seq database (GSE290654) revealed that H3K18la is significantly enriched near transcription start sites genome‐wide (Figure 3E–G). Through integrative genomic viewer visualization, we identified an extremely prominent H3K18la enrichment peak upstream of the RPL11 gene promoter (‐1500 to −1000 bp, chr4: 135776100–135776250) (Figure 3H). Our ChIP‐qPCR assays performed in GEM‐R cell lines further confirmed that, compared to parental sensitive cells, the H3K18la occupancy level at the RPL11 promoter region (specifically the P3 region, −1500 to −1000 bp) was significantly elevated in resistant cells (Figure 3I). Furthermore, we found that the acetyltransferase EP300, acting as a crucial “writer” that catalyzes histone lactylation, also exhibited significantly enhanced enrichment at the same promoter region in resistant cells (Figure 3J). This indicates that driven by lactatic metabolic reprogramming, EP300 is recruited to the RPL11 promoter to catalyze H3K18 lactylation, which in turn mediates the transcriptional activation of RPL11.

To directly test whether lactylation at the H3K18 site is a necessary condition for RPL11 transcriptional activation, we introduced a specific H3K18 site mutant (H3K18R, mutating lysine to an unlactylatable arginine) for loss‐of‐function experiments. Dual‐luciferase reporter assays showed that in cells co‐transfected with wild‐type histone H3 (H3‐WT), lactate treatment significantly enhanced RPL11 promoter activity; however, in the H3K18R mutant group, this lactate‐driven transcriptional activation was almost completely abrogated (Figure S3A,C). Synchronous IF results also demonstrated that the H3K18R mutation effectively neutralized lactate‐induced nuclear translocation of RPL11 (Figure S3B,D). Furthermore, we treated GEM‐R cells with an LDHA inhibitor (to block lactate production at the source) and A‐485 (to specifically inhibit the catalytic function of EP300). The results revealed that these two upstream targeted interventions not only significantly diminished H3K18la enrichment at the RPL11 promoter but also led to a sharp and synchronous decline in RPL11 expression at both the mRNA and protein levels (Figure S3E–J). These data further definitively establish that specific deposition of H3K18la is a necessary prerequisite for activating RPL11 transcription.

Finally, we explored the ultimate output effect of the lactate‐H3K18la‐RPL11 axis on ribosomal function and global protein translation. The results demonstrated that the lactate‐driven pathway significantly promoted the transcription of 18S and 28S pre‐rRNAs, whereas blocking this pathway with LDHi suppressed this process (Figure 3K,L and Figure S2L). SUnSET assays based on puromycin incorporation visually confirmed that lactate treatment drastically enhanced the global protein synthesis rate of the cells, while inhibiting lactylation led to a severe attenuation of this synthetic capacity (Figure 3M,N and Figure S2M). In summary, this study reveals that aberrantly accumulated lactate signaling drives the enrichment of EP300‐mediated H3K18la modification at the RPL11 promoter, which acts as a causal mechanism to directly activate RPL11 transcription; this in turn systematically enhances ribosome biogenesis and global protein translation capacity, ultimately endowing PDAC cells with a powerful survival advantage against chemotherapeutic stress.

### 2.4 RPL11 Is a Pivotal Functional Hub That Mediates GEM Resistance and Malignant Phenotypes in PDAC

Based on the aforementioned discovery of the mechanism by which H3K18la regulates RPL11 transcription, we further explored the exact biological function of RPL11 in mediating acquired GEM resistance in PDAC. First, we evaluated the expression dynamics of RPL11 in in vivo and in vitro acquired resistance models. In xenograft tumors subjected to cyclic in vivo GEM screening and continuous passaging, the mRNA and protein expression levels of RPL11 were progressively upregulated with the establishment of the resistant phenotype (Figure 4A,B). Consistently, in resistant cell lines established in vitro through stepwise dose escalation of GEM, RPL11 expression also exhibited a significant time‐dependent increase (Figure 4C). Furthermore, analysis of a public preclinical database (GSE140077) confirmed that RPL11 transcription levels in GEM‐R PDAC cells were significantly higher than those in their sensitive parental counterparts (Figure 4D). These consistent data indicate that high RPL11 expression is a robust molecular hallmark of acquired GEM resistance in PDAC.

**FIGURE 4:**
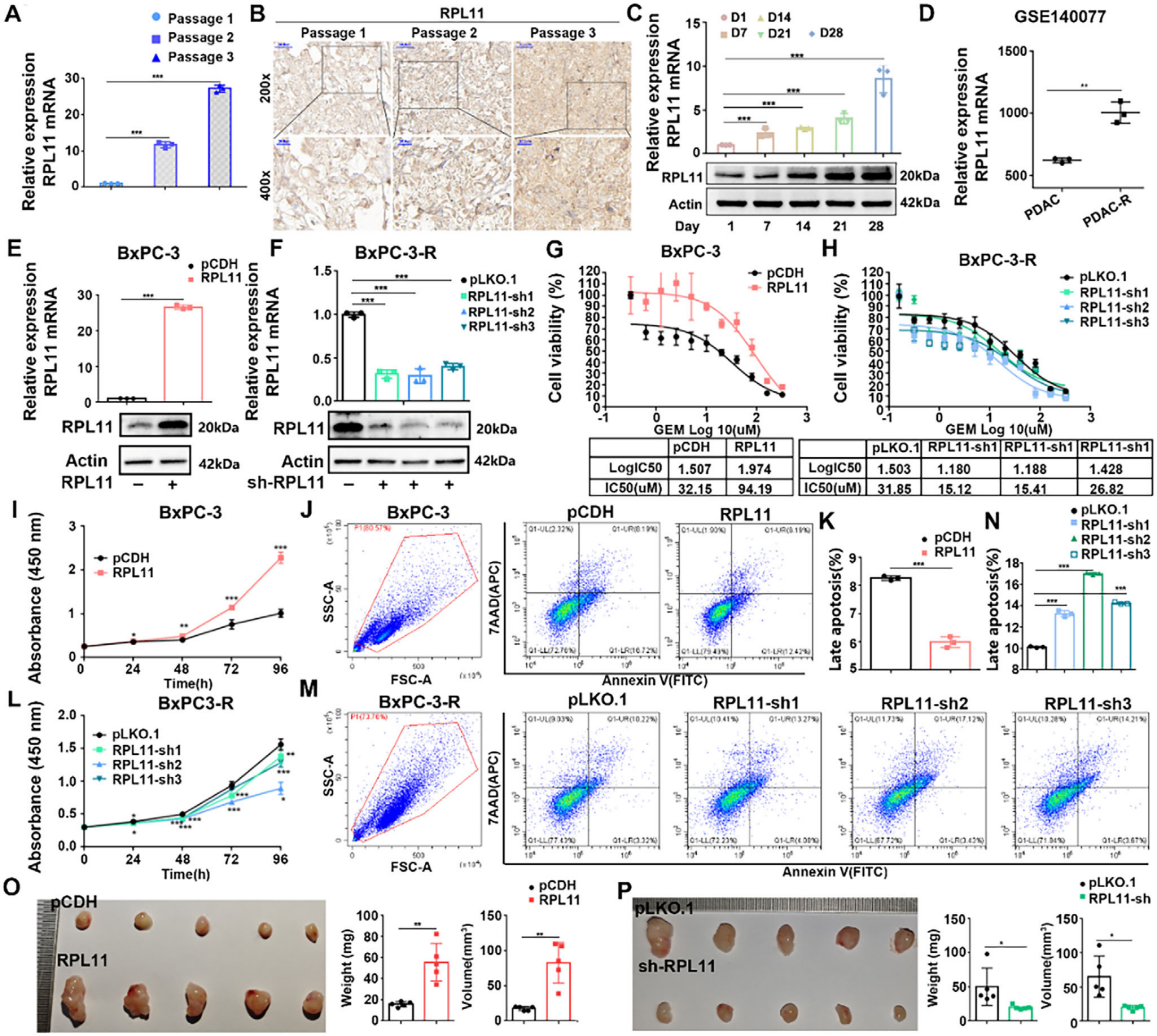
RPL11 upregulation serves as a key driver of acquired gemcitabine resistance and promotes malignant progression in PDAC. (A,B) Gradual increases in RPL11 (A) mRNA and (B) protein levels across passages (Passages 1–3) in an in vivo BxPC‐3 acquired resistance model induced by cyclic GEM screening. (A) RT‐qPCR analysis; (B) Representative IHC staining images, scale bars: 50 µm (200×, top) and 20 µm (400×, bottom). (C) Synchronous upregulation of RPL11 mRNA and protein over time (days 1–28) in an in vitro continuous GEM‐induced resistance model (BxPC‐3‐R). (D) Analysis of the GEO database (GSE140077) confirms significantly higher RPL11 mRNA expression in PDAC‐resistant (PDAC‐R) clones compared to parental cells. (E,F) RT‐qPCR and Western blot validation of stable RPL11 overexpression (OE) and knockdown (sh‐RPL11) efficiency in BxPC‐3 and BxPC‐3‐R cells. pCDH and pLKO.1 served as an empty vector controls. (G,H) Dose‐response cell viability curves and IC50 values evaluating the effects of (G) RPL11 OE on GEM sensitivity in BxPC‐3 cells and (H) RPL11 knockdown on GEM resistance in BxPC‐3‐R cells. (I–N) RPL11 promotes cell proliferation and inhibits apoptosis. (I,L) CCK‐8 assays showing that RPL11 OE promotes BxPC‐3 proliferation (I), whereas RPL11 knockdown suppresses BxPC‐3‐R proliferation (L). (J,K,M,N) Flow cytometry analysis (Annexin V/7‐AAD). RPL11 OE inhibits late apoptosis in BxPC‐3 cells (J,K), whereas RPL11 knockdown promotes late apoptosis in BxPC‐3‐R cells (M,N). Representative scatter plots (J,M) and quantification of late apoptosis rates (K,N) are shown. (O,P) In vivo evaluation in BxPC‐3 subcutaneous xenograft models showing that RPL11 OE promotes tumor growth, whereas its knockdown inhibits growth. Representative endpoint tumor images (O) and quantification of tumor weights and volumes (P) are shown (n = 5/group). Data are presented as mean ± SD (in vitro *n* = 3). Statistical significance was evaluated by two‐tailed Student's t‐test. ^*^p < 0.05, ^**^p < 0.01, ^***^p < 0.001.

To establish a causal relationship between RPL11 and chemoresistance, we performed rigorous bidirectional intervention experiments across distinct cellular genetic backgrounds. In “gain‐of‐function” experiments, we stably overexpressed RPL11 in parental (GEM‐sensitive) PDAC cell lines (BxPC‐3, PANC‐1, and Panc02) (Figure 4E and Figure S4A,K). Drug sensitivity analysis revealed that the introduction of exogenous RPL11 significantly increased the IC50 of GEM, directly conferring chemoresistance to the parental cells (Figure 4G and Figure S4C,M). Conversely, to ascertain whether targeting RPL11 could truly overcome established resistance, we conducted “loss‐of‐function” experiments in GEM‐R cell lines (Figure 4F and Figure S4B,L). The results clearly demonstrated that knocking down RPL11 via shRNA substantially reduced the IC50 values of GEM‐R cells. This effect represents a substantial reversal of the resistant phenotype (resensitization) rather than a general additive cytotoxicity (Figure 4H and Figure S4D,N).

To further evaluate whether this mechanism is universally applicable to broad‐spectrum chemotherapeutics, we tested the impact of altered RPL11 expression on the sensitivity of PDAC to other first‐line clinical standard treatments. The results showed that RPL11 overexpression significantly enhanced the cross‐tolerance of parental cells to DNA damage‐inducing chemotherapeutics, such as oxaliplatin and 5‐fluorouracil (5‐FU) (Figure S5A,E). Correspondingly, knocking down RPL11 in GEM‐R cells decreased the IC50 of these drugs, effectively reversing the cross‐resistance (Figure S5B,F). IF staining for γH2AX confirmed that after 24 h of oxaliplatin treatment, the number of DNA double‐strand break foci was significantly reduced in RPL11‐overexpressing cells, whereas RPL11 knockdown led to a massive accumulation of damage foci (Figure S5C,D), suggesting that RPL11‐enhanced DNA damage repair capacity is the core mechanism underlying this cross‐resistance. Interestingly, RPL11 overexpression or knockdown also significantly modulated sensitivity to irinotecan (a topoisomerase I inhibitor) (Figure S5G,H), a result highly consistent with recent literature reports [20]. However, there was no significant impact on the sensitivity to paclitaxel (a microtubule stabilizer) (Figure S5I,J), indicating that the drug selectivity of this resistance axis is not indiscriminate but primarily targets chemotherapeutics whose main pharmacological mechanism involves DNA damage.

Beyond regulating chemosensitivity, RPL11 also plays a central role in maintaining the malignant biological behaviors of PDAC cells. CCK‐8 proliferation assays and Annexin V/PI flow cytometry analysis revealed that RPL11 overexpression significantly accelerated cell proliferation (Figure 4I and Figure S4E,O) and effectively reduced the basal cell apoptosis rate (Figure 4J,K and Figure S4F,G,P,Q). Conversely, knocking down RPL11 in GEM‐R cells severely impaired their in vitro proliferation capacity (Figure 4L and Figure S4H,R) and drastically induced cellular apoptosis (Figure 4M,N and Figure S4I,J,S,T). To confirm these findings in vivo, we constructed xenograft tumor models using human (BxPC‐3) and murine (Panc02) PDAC cell lines combined with a high‐concentration Matrigel matrix. In vivo experiments confirmed that under this microenvironmental pressure, RPL11 overexpression significantly accelerated tumor growth kinetics, resulting in a substantial increase in tumor volume and weight (Figure 4O and Figure S4U). In contrast, targeted knockdown of RPL11 extremely effectively suppressed the in vivo progression of PDAC xenografts under conditions simulating the authentic microenvironment (Figure 4P and Figure S4V). Taken together, these data demonstrate that RPL11 is not only a key molecule driving the malignant proliferation and apoptotic evasion of PDAC cells but also the core functional hub maintaining the acquired GEM‐R phenotype.

### 2.5 RPL11 Drives PDAC Resistance by Post‐translationally Stabilizing EEF1A1 to Enhance Global Protein Synthesis

To systematically elucidate the molecular mechanisms underlying RPL11‐driven GEM resistance in PDAC, we performed ribosome profiling (Ribo‐seq) in RPL11‐knockdown Panc02‐R cells. Gene Ontology (GO) enrichment analysis revealed that RPL11 depletion significantly suppressed biological processes such as “translational elongation” and “ribosome biogenesis” (Figure 5A,B). Through in‐depth analysis of genes with differential translation efficiency, we identified EEF1A1 as one of the most significantly downregulated candidate targets (Figure 5C). Codon occupancy analysis further demonstrated that RPL11 loss led to significant ribosome stalling near the stop codon (Figure 5D), indicative of impaired translational elongation. To corroborate these findings, we conducted polysome profiling and SUnSET assays. The results showed that RPL11 overexpression in BxPC‐3 and Panc02 cells significantly increased the polysome‐to‐monosome (P/M) ratio and enhanced the global protein synthesis rate (Figure 5E,F). Conversely, RPL11 knockdown led to a decreased P/M ratio and significantly inhibited puromycin incorporation in BxPC‐3‐R and Panc02‐R cells (Figure 5G,H). Together, these genome‐wide and functional lines of evidence demonstrate that RPL11 is a crucial factor for maintaining highly efficient translational elongation and global protein synthesis flux in PDAC cells.

**FIGURE 5:**
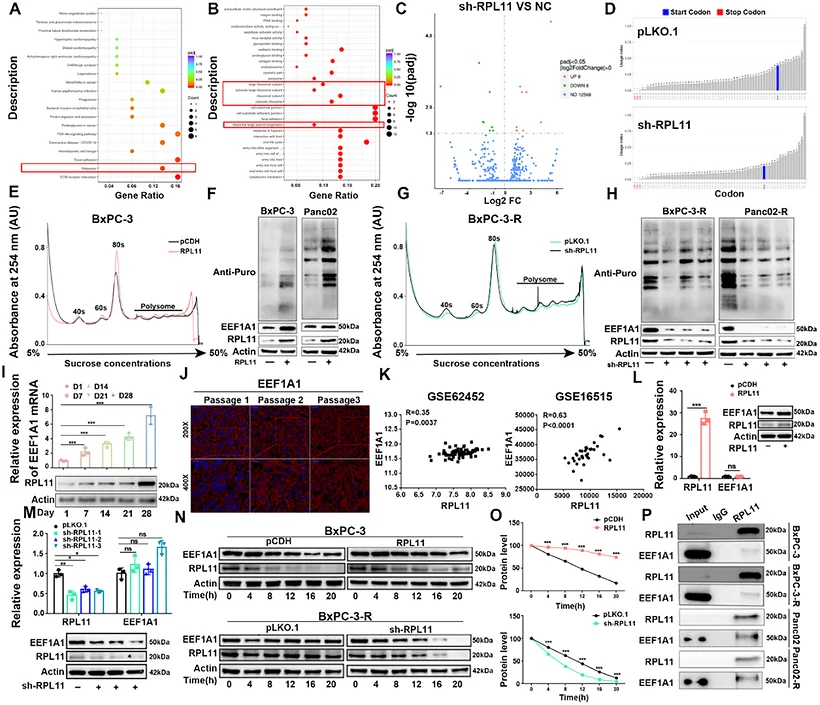
Cytoplasmic RPL11 drives global protein synthesis by interacting with EEF1A1 and enhancing its stability. (A‐D) Ribosome profiling (Ribo‐seq) reveals that RPL11 primarily regulates translation elongation. (A,B) GO enrichment analysis in sh‐RPL11 Panc02‐R cells indicates that the suppressed processes predominantly involve translation initiation and elongation. (C) Volcano plot of translation efficiency (TE) identifying elongation factor EEF1A1 as a critical downregulated gene upon RPL11 knockdown. (D) Codon occupancy analysis showing increased ribosome stalling at stop codons in RPL11‐knockdown cells compared to the pLKO.1 control. (E–H) Polysome profiling and SUnSET assays confirming that RPL11 positively regulates global protein synthesis. RPL11 OE significantly increases the polysome‐to‐monosome ratio (E) and puromycin‐labeled global protein synthesis rates (F) in BxPC‐3 and Panc02 cells. Conversely, RPL11 knockdown decreases these metrics in BxPC‐3‐R and Panc02‐R cells (G,H). (I–P) RPL11 positively regulates EEF1A1 at the post‐translational level via direct physical interaction. (I) Synchronous, time‐dependent upregulation of EEF1A1 mRNA and protein during in vitro GEM‐induced resistance. (J) Increasing EEF1A1 protein levels (red) across passages in the in vivo cyclic screening model. Scale bars: 50 µm (200×, top) and 20 µm (400×, bottom). (K) Pearson correlation analysis of PDAC patient cohorts (GSE62452, GSE16515) showing a significant positive correlation between RPL11 and EEF1A1 mRNA levels. (L,M) RT‐qPCR and Western blot demonstrating that RPL11 OE (L) or knockdown (M) in BxPC‐3 cells significantly alters EEF1A1 protein levels without affecting mRNA levels (ns). (N,O) Cycloheximide (CHX) chase assays demonstrating that RPL11 OE prolongs the half‐life of EEF1A1, whereas sh‐RPL11 shortens it. Quantification results are shown on the right. (P) Endogenous co‐immunoprecipitation (Co‐IP) confirming the physical interaction between RPL11 and EEF1A1 across multiple PDAC cell lines. Data are presented as mean ± SD (n = 3). Statistical significance was evaluated by a two‐tailed Student's t‐test. ns: no significance, ^*^p < 0.05, ^**^p < 0.01, ^***^p < 0.001.

We next investigated the molecular mode by which RPL11 regulates EEF1A1 expression. In the in vivo GEM resistance evolution model, the protein levels of RPL11 and EEF1A1 exhibited synchronized upregulation (Figure 5I,J). Interestingly, although public databases (GSE62452, GSE16515) indicated a significant positive correlation between their mRNA levels in PDAC patient cohorts (Figure 5K), in vitro experiments showed that modulating RPL11 levels only induced dramatic changes in EEF1A1 protein abundance, with minimal impact on its mRNA levels (Figure 5L,M). This apparent discrepancy suggests that the mRNA correlation observed in clinical samples may stem from common upstream microenvironmental drivers (such as high lactate‐induced co‐transcription), whereas at the molecular level, the direct regulation of EEF1A1 by RPL11 predominantly occurs at the post‐translational stage. Furthermore, cycloheximide (CHX) chase assays confirmed that RPL11 overexpression significantly prolonged the protein half‐life of EEF1A1, whereas RPL11 knockdown accelerated its degradation (Figure 5N,O). Co‐IP assays explicitly verified the physical interaction between endogenous RPL11 and EEF1A1 across various PDAC cell lines (Figure 5P). These data reveal that RPL11 upregulates EEF1A1 expression at the post‐translational level by directly binding to it and enhancing its protein stability.

Subsequently, to evaluate the independent function of EEF1A1 in PDAC, we performed gain‐ and loss‐of‐function experiments in parental (GEM‐sensitive) and GEM‐R cells (Figure S6A,B). The results demonstrated that overexpressing EEF1A1 in parental cells significantly elevated their resistance to GEM (reflected by an increased IC50) and conferred proliferative advantages and anti‐apoptotic capabilities (Figure S6C,E–G,M,O–Q). Conversely, knocking down EEF1A1 in GEM‐R cells achieved a substantial reversal of the resistant phenotype (significantly enhancing chemosensitivity) and severely impaired malignant proliferation and survival (Figure S6D, H–J,N,R–T). These results position EEF1A1 as a core functional molecule driving chemoresistance and malignant progression in PDAC.

Finally, to ascertain whether EEF1A1 is a necessary mediator for RPL11 function, we designed bidirectional rescue experiments (Figure S7A,B). The results showed that silencing EEF1A1 in parental cells completely abolished the remodeling of the translational machinery and the surge in global protein synthesis induced by exogenous RPL11 overexpression (Figure 6A,B and Figure S7C). This blockade of translational capacity directly abrogated the newly acquired resistance and survival advantage conferred by RPL11, and the accelerated proliferation and apoptosis evasion under chemotherapy stress were completely reversed (Figure 6E,F,I and Figure S7D,E,H).

**FIGURE 6:**
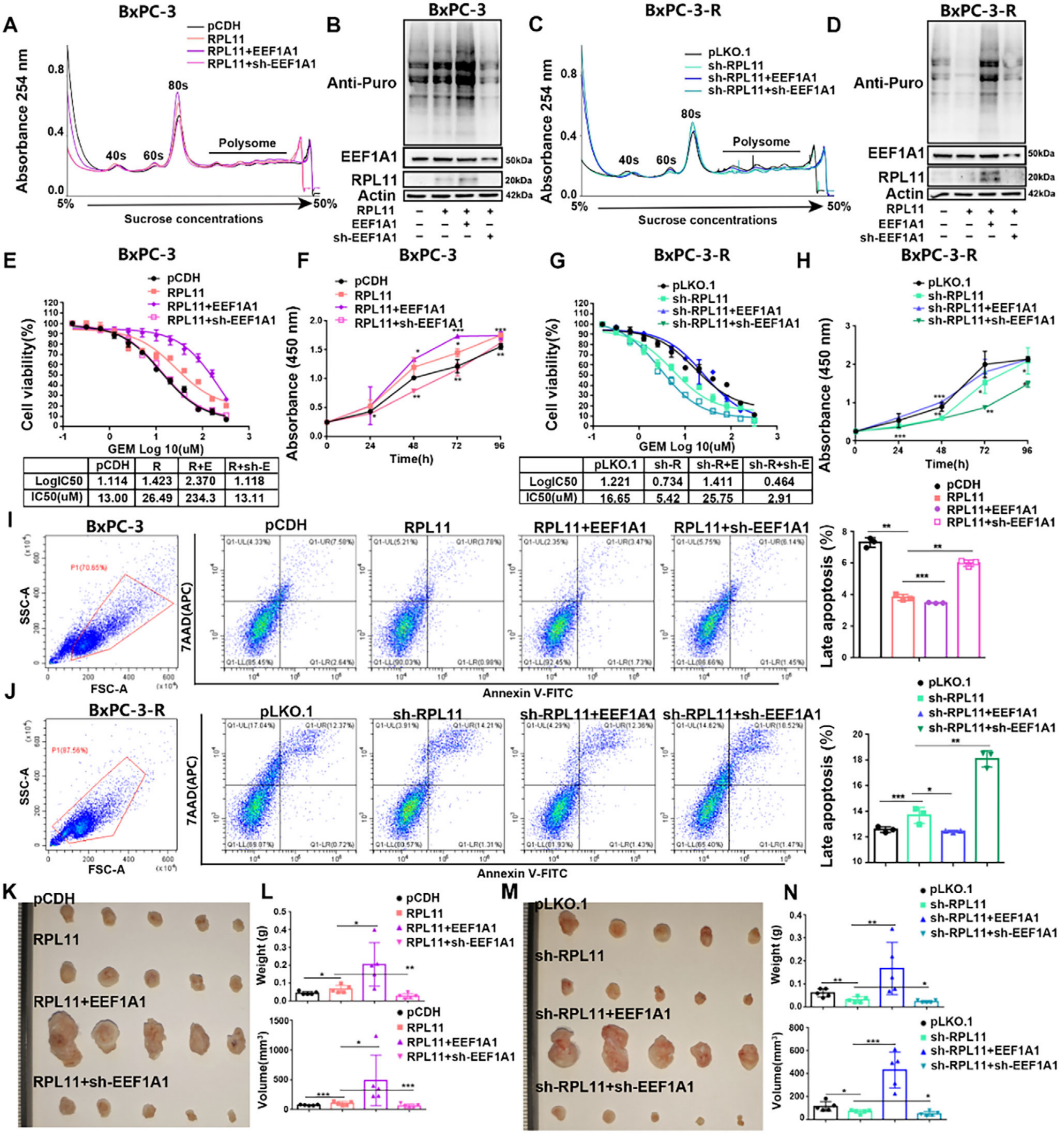
The oncogenic and chemoresistant phenotypes mediated by RPL11 strictly depend on its downstream effector EEF1A1. (A,B,E,F, I,K,L) EEF1A1 knockdown significantly antagonizes RPL11‐driven translational enhancement and malignant phenotypes. Polysome profiling (A) and SUnSET assays (B) show that EEF1A1 knockdown reverses RPL11 OE‐induced global translational activation (right: Western blot validation). Cell viability (E) and proliferation (F) curves indicate that EEF1A1 knockdown resensitizes RPL11‐OE cells to GEM and suppresses their proliferation. (I) Flow cytometry confirms that EEF1A1 knockdown abolishes RPL11‐mediated anti‐apoptotic effects. Nude mouse xenograft models validate that EEF1A1 knockdown significantly attenuates RPL11‐driven in vivo tumor growth (K,L) (*n* = 5/group). (C,D,G,H,J,M,N) Ectopic restoration of EEF1A1 rescues translation inhibition and phenotypic defects induced by RPL11 deficiency. Polysome profiling (C) and SUnSET assays (D) show that expressing EEF1A1 in sh‐RPL11 cells repairs impaired global translation rates. Viability (G) and proliferation (H) curves demonstrate that EEF1A1 restoration significantly reverses GEM sensitization and growth arrest induced by RPL11 knockdown. (J) Flow cytometry shows that EEF1A1 restoration antagonizes sh‐RPL11‐induced apoptosis. In vivo validation confirms that restoring EEF1A1 effectively rescues tumor growth suppression caused by RPL11 deficiency (M,N) (*n* = 5/group). Data are presented as mean ± SD (in vitro *n* = 3). Statistical significance was evaluated by two‐tailed Student's t‐test. ^*^p < 0.05, ^**^p < 0.01, ^***^p < 0.001.

Reversely, in RPL11‐knockdown GEM‐R cells, the exogenous introduction of EEF1A1 successfully “bypassed” the dependency on RPL11, largely compensated for the translational defects, and reactivated global protein synthesis (Figure 6C,D and Figure S7C), fully manifesting as a “comprehensive resurgence” of chemoresistance (Figure 6G,H,J and Figure S7F,G,I). Evaluation using an in vivo xenograft tumor model revealed that the depletion of EEF1A1 completely abolished the accelerated tumor progression and resistant growth driven by RPL11 overexpression in vivo (Figure 6K,L). In contrast, compensatory expression of EEF1A1 in RPL11‐depleted tumors successfully rescued tumor growth arrest (Figure 6M,N), further validating this strict dependency.

In summary, this study uncovers a definitive “RPL11‐EEF1A1‐global translation” signaling axis, establishing the core mechanism by which RPL11 maintains high‐throughput translation by post‐translationally stabilizing EEF1A1, thereby conferring acquired chemoresistance to PDAC cells.

### 2.6 Nuclear RPL11 Drives GEM Resistance by Transcriptionally Activating PARP14 to Enhance DNA Damage Repair

Given that the core anti‐tumor mechanism of GEM relies on inducing lethal DNA damage [19], we hypothesized that the massive nuclear accumulation of RPL11 during the evolution of chemoresistance might directly participate in the reprogramming of the DNA damage response (DDR). To systematically identify the nuclear transcriptional targets of RPL11, we performed RNA‐seq analysis on RPL11‐knockdown BxPC‐3‐R resistant cells. Within the significantly downregulated gene network, poly(ADP‐ribose) polymerase 14 (PARP14) [20], a key effector molecule in the DDR pathway, emerged as a core candidate target (Figure 7A). Subsequently, loss‐ and gain‐of‐function validations performed across multiple PDAC cell lines via Western blotting and qPCR consistently confirmed that RPL11 significantly and positively regulates PARP14 expression at both the mRNA and protein levels (Figure 7B and Figures S8A,B and S12A). Furthermore, in both in vitro and in vivo acquired resistance models, the expression of PARP14 and RPL11 exhibited highly synchronized upregulation (Figure 7C,D and Figure S12B). Subcellular localization analysis further revealed that under lactate exposure or GEM resistance stress, their co‐localization and accumulation within the nucleus were significantly enhanced (Figure 7E,F and Figure S12C,D). Crucially, ChIP‐qPCR assays confirmed that nuclear RPL11 specifically binds to the P3 region (−1500 to −1000 bp) of the PARP14 promoter (Figure 7G and Figure S8C,D), exerting a direct transcriptional co‐activation function.

**FIGURE 7:**
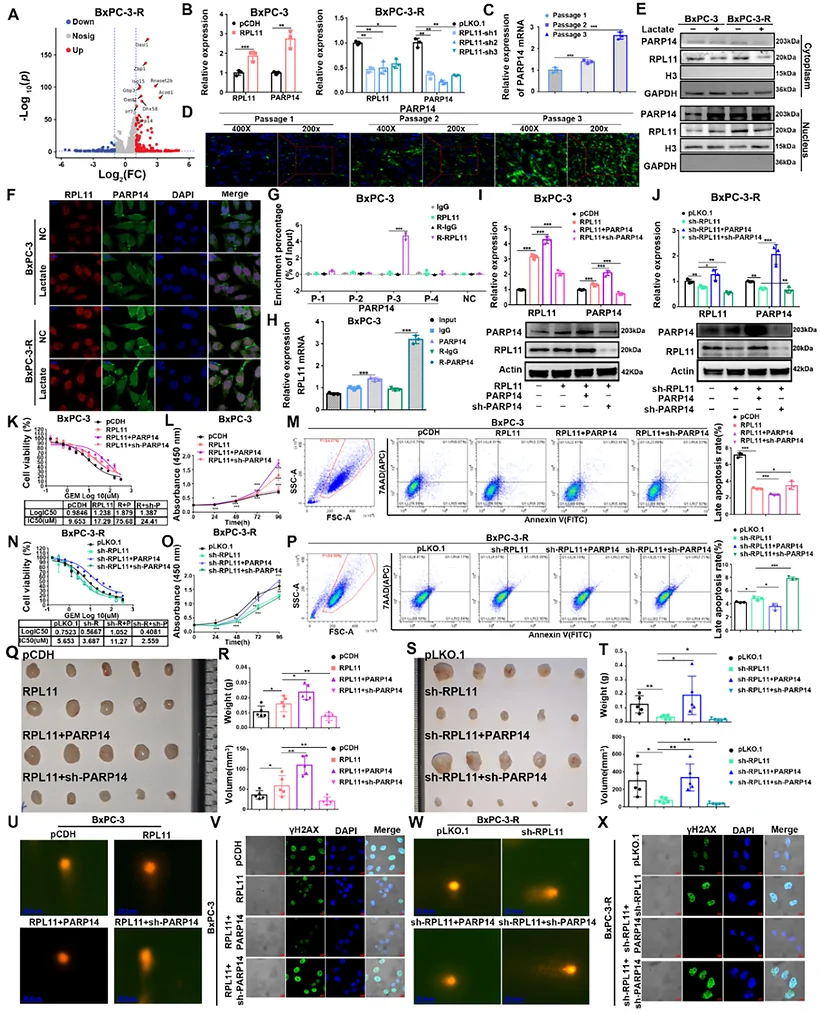
Nuclear RPL11 transcriptionally activates PARP14 to enhance DNA damage repair and mediate gemcitabine resistance. (A–F) RPL11 positively regulates PARP14, with both synchronously upregulated and co‐enriched in the nucleus of resistant models. (A) RNA‐seq volcano plot identifies PARP14 as a key downregulated DNA damage repair gene following RPL11 knockdown. (B) RT‐qPCR validation of PARP14 mRNA regulation by RPL11 OE or sh‐RPL11. (C,D) Progressive upregulation of PARP14 mRNA (C) and protein (D) across passages in the in vivo Panc02 cyclic resistance model. (E) Nuclear‐cytoplasmic fractionation confirms synchronous nuclear accumulation of RPL11 and PARP14 under lactate stimulation or in the resistance state. (F) IF staining demonstrating strong nuclear spatial colocalization of RPL11 (red) and PARP14 (green). Scale bar: 60 µm. (G,H) RPL11 regulates PARP14 by binding its promoter and mRNA. (G) ChIP‐qPCR showing specific enrichment of RPL11 protein at the PARP14 promoter (P3 site); (H) RNA immunoprecipitation (RIP‐qPCR) revealing direct binding of RPL11 to PARP14 mRNA. (I–T) Genetic rescue experiments confirming that RPL11‐mediated chemoresistance depends strictly on PARP14. (I–P) In vitro functional validation (gene intervention efficiency shown in I, J): PARP14 knockdown reverses GEM resistance (K), anti‐proliferation (L), and anti‐apoptosis (M) in RPL11‐OE cells; conversely, PARP14 overexpression rescues the phenotypic defects of sh‐RPL11 cells (N–P). (Q–T) In vivo validation in xenograft models (*n* = 5/group): RPL11‐driven tumor growth is significantly suppressed by PARP14 knockdown (Q,R), while sh‐RPL11‐induced growth inhibition is rescued by PARP14 restoration (S,T). (U–X) RPL11 enhances DNA damage repair efficacy via PARP14. (U,V) Comet assays (200×, 20 µm) and γH2AX IF staining (Scale bar: 10 µm) show that PARP14 knockdown impairs RPL11‐mediated DNA repair, exacerbating DNA damage. (W,X) Restoring PARP14 in sh‐RPL11 cells effectively promotes DNA repair and reduces γH2AX foci formation. Data are presented as mean ± SD (*n* = 3 independent experiments). Statistical significance was evaluated by a two‐tailed Student's t‐test. ^*^p < 0.05, ^**^p < 0.01, ^***^p < 0.001.

Since RPL11 lacks a classic nuclear localization signal, yet its nuclear accumulation is a prerequisite for activating PARP14 transcription, we further investigated the mechanism of its nuclear import and its necessity for resistance. RPs are reported to rely on non‐classical nuclear import pathways, often involving Importin family members, for their entry into the nucleus to participate in ribosome biogenesis [21]. Importin 7 (IPO7) has been identified as a mediator of RPL11 nuclear import [22]. To verify the functional relevance of this pathway under lactate/resistance stress, we treated lactate‐stimulated or GEM‐R PDAC cells with Importazole, a specific Importin‐β pathway inhibitor. IF co‐localization analysis demonstrated that the nuclear RPL11 signal virtually disappeared following Importazole treatment (Figure S8E). Nuclear/cytoplasmic fractionation combined with Western blot analysis also showed that Importazole treatment significantly blocked the nuclear accumulation of RPL11, resulting in its cytoplasmic retention (Figure S8F). This blockade of nuclear transport directly impaired the recruitment and transcriptional co‐activation of RPL11 at the PARP14 promoter, thereby largely reversing the GEM‐R phenotype of the cells (Figure S8G–I).

Further exploration of the upstream regulation revealed that exogenous lactate treatment synchronously increased H3K18la enrichment at the PARP14 promoter region and enhanced RPL11 recruitment to this site; conversely, treatment with the LDHA inhibitor (Oxamate) abolished this process (Figure S8J,K). This demonstrates that H3K18la‐mediated chromatin remodeling is a prerequisite for recruiting nuclear RPL11 to the PARP14 promoter.

Additionally, based on the RIP‐qPCR finding that RPL11 interacts with PARP14 pre‐mRNA (Figure 7H and Figure S11A), we conducted an Actinomycin D (ActD) chase assay. The data clearly showed that RPL11 significantly prolonged the half‐life of PARP14 mRNA (Figure S11B,C). Taken together, nuclear RPL11 exhibits a dual regulatory mechanism: it is recruited by H3K18la to the promoter to act as a transcriptional co‐activator driving transcription, while also binding to the mRNA post‐transcriptionally to enhance its stability.

Having established this regulatory relationship, we independently evaluated the clinical significance and biological function of PARP14 in PDAC. Data mining from clinical cohorts confirmed that PARP14 is significantly highly expressed in PDAC tissues (Figure S9A–D), and its high abundance is closely correlated with poor patient prognosis (Figure S9E–G), indicating important clinicopathological significance. At the functional level, ectopic overexpression of PARP14 in sensitive parental cells directly conferred robust chemoresistance and obvious malignant proliferative advantages (Figures S9H,J–M and S10A–C,G–I,M,O). In contrast, targeted knockdown of PARP14 in GEM‐R cells substantially abolished GEM tolerance, achieving a reversal of the resistant phenotype (Figures S9I,N–Q and S10D–F,J–L,N,P). In vivo xenograft models also fully corroborated the pro‐tumorigenic role of PARP14 within the complex TME (Figure S9R,S). These data unambiguously position PARP14 as a core effector molecule driving chemoresistance in PDAC.

Next, to definitively ascertain whether the nuclear function of RPL11 is strictly dependent on PARP14, we designed and executed a series of critical rescue experiments. The results demonstrated that knocking down PARP14 in parental cells completely neutralized the GEM resistance and anti‐apoptotic survival advantages conferred by exogenous RPL11 overexpression (Figure 7I,K–M and Figure S11D,F–H and S12E,G–I). Reversely, in RPL11‐depleted GEM‐R cells that had regained chemosensitivity, the exogenous re‐introduction of PARP14 was sufficient to compensate for the functional defects caused by RPL11 loss, enabling the cells to reacquire robust resistance to GEM (Figure 7J,N–P and Figures S11E,I–K and Figure S12F,J–L). Similarly, the xenograft tumor model established using human BxPC‐3 cells further confirmed that PARP14 depletion completely blocked the accelerated in vivo tumor progression driven by RPL11 overexpression (Figure 7Q,R), whereas PARP14 complementation effectively rescued the tumor growth arrest induced by RPL11 knockdown (Figure 7S,T). These results indicate that PARP14 is a critical and indispensable downstream effector mediating the nuclear pro‐resistance function of RPL11.

Based on the above findings, we further explored the biological essence of how this regulatory axis drives resistance. Alkaline comet assays and γH2AX IF staining visually demonstrated that in RPL11‐overexpressing cells, knocking down PARP14 led to a profound exacerbation of GEM‐induced DNA damage (evidenced by longer comet tails and persistent γH2AX retention), indicating a collapse of repair capacity (Figure 7U,V and Figures S11L,M and S12M,N). Conversely, restoring PARP14 expression in RPL11‐knockdown cells rapidly cleared DNA damage foci and reinstated efficient DDR capabilities (Figure 7W,X and Figures S11N,O and S12O,P). Therefore, we postulated that targeting the PARP family could serve as a breakthrough strategy to overcome resistance in PDAC with high RPL11 expression.

To this end, we introduced Olaparib (PARPi), an FDA‐approved clinical first‐line PARP inhibitor. In vitro drug susceptibility assays revealed that in high‐RPL11 expressing GEM‐R resistant cells, the combination of Olaparib and GEM produced a strong synergistic lethal effect, triggering massive apoptosis in the resistant cells (Figure S13A–F). Crucially, in our humanized xenograft tumor model, this combination regimen exhibited exceptional in vivo tumor growth inhibition without inducing significant systemic organ toxicity (Figure S13G–I).

In conclusion, this study not only unveils the “functional compartmentalization” of RPL11 from the cytoplasm to the nucleus and the comprehensive landscape by which it drives chemoresistance, but also provides a highly promising “drug repurposing” combination therapy strategy for PDAC patients who are resistant to GEM due to high lactate/high RPL11 expression, by introducing a clinically available therapeutic agent.

### 2.7 Co‐targeting the Lactate‐RPL11 Axis Synergistically Sensitizes PDAC to GEM and Potently Inhibits Disease Progression

Given that single‐target blockade in highly heterogeneous PDAC often readily triggers compensatory resistance [1], we proposed a combinatorial targeting strategy spanning from the metabolic source to the epigenetic/translational execution terminals. We systematically evaluated the synergistic interventional effects of simultaneously inhibiting lactate production (using LDHi) and blocking ribosome biogenesis (using CX‐5461). The results showed that in BxPC‐3‐R (Figure 8A) and Panc02‐R (Figure S14A) cell lines, which feature acquired resistance, although monotherapies exhibited only limited anti‐tumor activity, the triple combination therapy of LDHi, CX‐5461, and GEM demonstrated remarkably profound synergistic cytotoxicity. Dose‐response curve analysis confirmed that the addition of either LDHi or CX‐5461 decreased the IC50 values of GEM in resistant cells to varying degrees, whereas the triple combination achieved the maximal reversal of chemoresistance (Figure 8B and Figure S14B). Flow cytometry analysis further corroborated that the apoptosis rate induced by the triple therapy was significantly higher than that of any single‐agent or dual‐combination regimen (Figure 8C,D and Figure S14C,D), highlighting the potent synergistic effect of this strategy in inducing the death of resistant cells.

**FIGURE 8:**
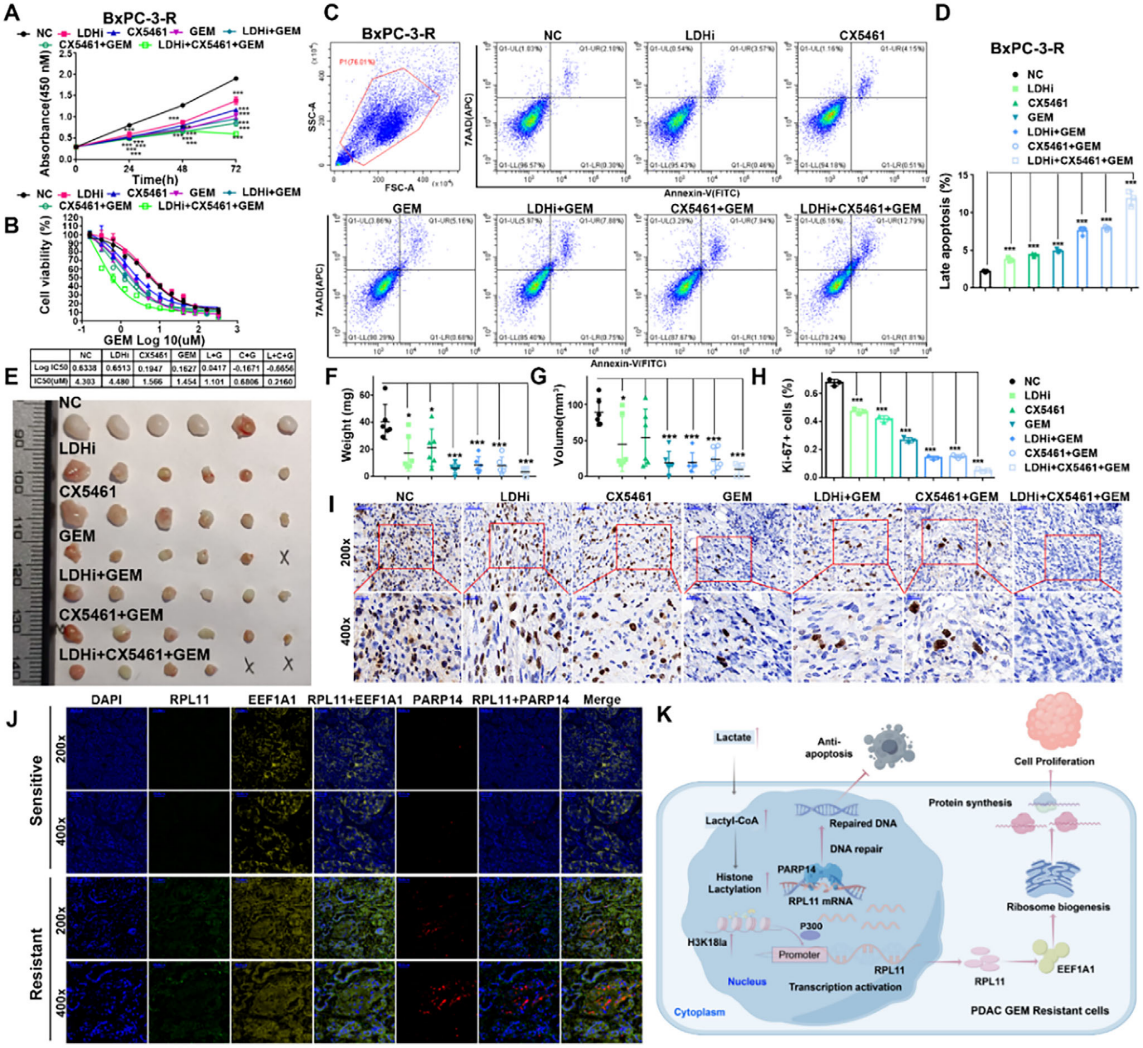
Combined targeting of lactate metabolism and ribosome biogenesis synergistically reverses gemcitabine resistance in PDAC. (A–D) Co‐treatment with LDHi and CX‐5461 significantly sensitizes cells to chemotherapy and induces apoptosis in vitro. (A,B) CCK‐8 assays evaluating the effects of combinatorial interventions (LDHA inhibitor LDHi, 10 µm; ribosome biogenesis inhibitor CX‐5461, 50 nm; alone or combined as pretreatment) on GEM sensitivity (A) and proliferation (B) in BxPC‐3‐R cells. The table below (B) lists the corresponding IC50 values. (C,D) Flow cytometry (Annexin V/7‐AAD) detecting apoptosis among groups. (C) Representative scatter plots; (D) Quantification of late apoptosis rates, demonstrating that the triple combination induces the most profound apoptosis. (E–I) The combination regimen exhibits robust synergistic anti‐tumor efficacy in vivo in BxPC‐3‐R xenograft models (*n* = 6/group). (E) Gross images of excised endpoint tumors; (F) Quantification of endpoint tumor weights; (G) Tumor volume growth curves indicating near‐complete suppression of tumor growth by the combination therapy. (H,I) IHC analysis and quantification of Ki67 positivity. Representative images scale bars: 50 µm (200×, top) and 20 µm (400×, bottom). (J) Clinical validation via multiplex immunofluorescence (mIF). Representative mIF images showing significantly higher fluorescence intensities and spatial co‐expression of RPL11 (green), EEF1A1 (yellow), and PARP14 (red) in GEM‐resistant PDAC tumor tissues compared to sensitive tissues. Scale bars: 50 µm (200×, top) and 20 µm (400×, bottom). (K) Schematic working model of the lactate‐H3K18la‐RPL11 signaling axis driving acquired gemcitabine resistance in PDAC. Data are presented as mean ± SD (in vitro *n* = 3). Statistical significance was evaluated by a two‐tailed Student's t‐test. ^*^p < 0.05, ^**^p < 0.01, ^***^p < 0.001.

To validate this strategy within a more complex in vivo microenvironment, we utilized human and murine xenograft tumor models that simulate the dense stroma. In vivo experiments revealed that, compared to monotherapy, the triple combination of LDHi + CX‐5461 + GEM yielded the most prominent anti‐tumor efficacy, leading to drastic shrinkage in tumor volume and weight (Figure 8E–G and Figure S14E–G). Immunohistochemistry (IHC) staining analysis showed that the Ki‐67 proliferation index in tumor tissues from the triple‐therapy group was reduced to the lowest level (Figure 8H,I), fully substantiating the in vivo effectiveness of the combinatorial regimen in curbing malignant proliferation. Importantly, throughout the treatment period, the body weights of the mice across all groups remained stable, and H&E staining of major organs (such as the liver and kidneys) revealed no obvious pathological lesions (Figure S14H,J). Additionally, key blood biochemical parameters (e.g., AST, ALT, CREA, UREA) were all maintained within the normal reference ranges (Figure S14I,K), indicating that this regimen possesses a highly favorable safety profile while delivering significant therapeutic efficacy.

Finally, to further evaluate the clinical relevance of the aforementioned mechanisms, we analyzed the expression patterns of key target proteins in a paired cohort of PDAC patients. Multiplex IF and IHC staining results demonstrated that, compared to chemosensitive tumor tissues, RPL11 expression was significantly upregulated in resistant tumor tissues (Figure 8J and Figure S14L,M). Simultaneously, its cytoplasmic effector protein EEF1A1 and nuclear effector protein PARP14 also exhibited profound synchronous upregulation (Figure 8J). Furthermore, correlation analysis based on clinical samples confirmed a significant positive correlation between the modification level of H3K18la and the protein abundance of RPL11 in PDAC tissues (Figure S14N). These data derived from clinical cohorts compellingly support and corroborate the molecular regulatory network unveiled in this study.

In summary, this study profoundly elucidates that in GEM‐R PDAC, excessive lactate driven by glycolytic reprogramming induces widespread H3K18la modification, which subsequently and specifically recruits the transcriptional machinery to activate the expression of the core ribosomal protein RPL11. The functionally activated RPL11 then ingeniously executes a spatial compartmentalization strategy: in the cytoplasm, it stabilizes the elongation factor EEF1A1 via direct protein‐protein interaction, forcefully driving global protein synthesis to sustain malignant proliferation; in the nucleus, functioning as a transcriptional co‐activator and mRNA stabilizer, it efficiently upregulates PARP14, thereby remodeling the DNA damage repair defense line (Figure 8K). This exquisite synergy of “metabolic sensing—epigenetic transduction—dual‐compartmental execution” confers an absolute survival advantage upon PDAC cells against devastating chemotherapeutic stress.

## 3 Discussion

Acquired resistance to GEM in PDAC is the fundamental cause of clinical treatment failure [23], yet the complex molecular network that drives this dynamic evolution remains incompletely understood. This study systematically uncovers a novel resistance trajectory in PDAC that connects metabolic reprogramming, epigenetic remodeling, and protein functional compartmentalization. We not only establish lactate as a key signaling molecule that specifically activates the transcription of the ribosomal protein RPL11 by driving H3K18la, but also, for the first time, elucidate an unprecedented “dual functional compartmentalization” mechanism for RPL11 in this specific genetic context—remodeling translation elongation flux in the cytoplasm while reprogramming the DDR in the nucleus. This work not only provides a new biological paradigm for understanding how tumors convert metabolic stress into a structural resistance defense but also offers a mechanism‐based, precision combination strategy to clinically overcome highly refractory PDAC.

The profound coupling of metabolism and epigenetics is a core upstream driver of PDAC resistance [24]. Aberrant glycolysis and a high‐lactate microenvironment are hallmark features of PDAC, but the precise role of lactate in chemoresistance has long been debated [3]. Recent studies have gradually shattered the traditional view of lactate as merely a “metabolic waste product,” repositioning it as an active epigenetic regulator [25]. In this study, we not only confirmed in clinical samples the significant enrichment of baseline lactate in tumors from resistant patients and its strong positive correlation with H3K18la, but also, through rigorous LDHA genetic knockdown and specific metabolite (but not acetate or pyruvate) rescue experiments, we have genetically established that lactate accumulation is a “necessary and sufficient” core trigger for acquired resistance. This finding imparts a critical therapeutic meaning to the classic “Warburg Effect”: high lactate is not just a product of metabolic adaptation but an active defense signal deployed by cells to cope with genotoxic stress, which directly reshapes the resistance gene expression profile through specific lactylation modifications [26]. Compared to previous studies that primarily focused on the role of lactylation in immunosuppression or tumor metastasis, our work is the first to establish a direct causal link between the “Lactate‐H3K18la” axis and the core clinical challenge of chemoresistance. This suggests that interventions targeting this pathway (e.g., targeting LDHA or the yet‐to‐be‐defined “lactylation writers and erasers”) hold immense potential for re‐sensitizing tumor cells to drugs.

Another central breakthrough of this study is the revelation of RPL11 as a key hub at the terminus of the “metabolic‐epigenetic” signaling cascade and its functional remodeling in the context of p53 mutation. Ribosomal proteins have traditionally been viewed as “housekeeping” components that maintain cellular homeostasis, but their non‐canonical functions are becoming a frontier in oncology research [27]. To clarify whether RPL11 is an indispensable mediator downstream of H3K18la, we demonstrated through a bypass exclusion experiment that in the absence of RPL11, even when exogenous lactate strongly induced widespread H3K18la enrichment, it completely failed to restore the resistant phenotype. This establishes RPL11 not merely as one of many effectors but as an irreplaceable central hub. Under normal physiological conditions, RPL11 acts as a “sensor” of cellular stress, executing tumor‐suppressive functions by activating the p53 pathway [28]. However, our research reveals that in PDAC, where p53 function is commonly compromised, this stress response pathway is “hijacked” by tumor cells. RPL11 undergoes a functional switch from a tumor suppressor to a pro‐survival hub, weaving an intricate resistance network by executing complementary functions in different subcellular compartments. In the cytoplasm, RPL11 systematically enhances global protein synthesis flux by stabilizing the translation elongation factor EEF1A1, providing a solid “material foundation” for resistant cells to weather the chemotherapy crisis. In the nucleus, through the synergistic combination of non‐classical Importin‐β‐mediated nuclear transport, lactate stress‐induced disruption of nucleolar assembly, and direct physical anchoring by H3K18la, RPL11 achieves efficient redistribution to chromatin. Acting as a transcriptional co‐activator, this non‐canonical function directly drives the expression of the key DDR protein PARP14, thereby precisely counteracting GEM‐induced DNA damage. This dual‐action mechanism—“sourcing” macromolecules in the cytoplasm and “defending” the genome in the nucleus, orchestrated by a single protein—overturns the traditional understanding of ribosomal proteins. This mechanism also perfectly explains the multi‐dimensional observations from both clinical and in vitro data: the positive correlation of RPL11 and EEF1A1 mRNA levels in clinical samples (originating from an upstream lactate/H3K18la‐driven co‐transcriptional module), whereas under controlled in vitro stress, RPL11 primarily regulates EEF1A1 stability at the post‐translational level through PPI.

From a translational medicine perspective, the combination therapy strategy proposed in this study holds high clinical feasibility and great promise. Notably, the DDR program reinforced by the RPL11‐PARP14 axis is not specific to GEM. Our data confirm that the “Lactate‐H3K18la‐RPL11” mechanism also confers profound cross‐resistance to a broad spectrum of first‐line DNA‐damaging agents, including oxaliplatin and 5‐fluorouracil, which perfectly explains the multi‐drug resistance phenomenon often observed in PDAC patients. Moreover, to rigorously distinguish between “mechanism‐based resistance reversal” and “non‐specific additive toxicity,” we moved beyond conventional models and innovatively established a Matrigel‐rich, humanized biomimetic xenograft model with BxPC‐3 cells. This model maximally recapitulates the pro‐proliferative, stroma‐rich, and high‐lactate physiological microenvironment of human PDAC. Under these stringent conditions, we demonstrated that co‐targeting with an LDHi (upstream) or CX‐5461 (downstream) in combination with GEM triggers profound synergistic lethality, leading to significant tumor regression with excellent tolerability. Critically, given the translational hurdles faced by many preclinical tool compounds, we prospectively incorporated Olaparib [29], an FDA‐approved first‐line PARP inhibitor, into a salvage intervention strategy. The in vivo data provide strong evidence that Olaparib can precisely block the downstream resistance pathway of RPL11‐PARP14 and produce deep synergy with GEM. This “drug repurposing” strategy directly bypasses the lengthy drug development cycle, offering a “plug‐and‐play” intervention blueprint for treating refractory PDAC. At the same time, high expression of H3K18la or RPL11 in tumor tissues holds great potential as reliable biomarkers for future precision stratification and for selecting patient populations who will benefit most from GEM and combination therapies.

Of course, the findings of this study also open up new avenues for future exploration. First, the precise upstream signals that drive the hyper‐glycolytic state of PDAC in its harsh microenvironment still need to be traced. Second, whether the lactate signal participates in broader transcriptional reprogramming through other modification sites or non‐histone substrates remains unknown. Mechanistically, how H3K18la precisely orchestrates chromatin remodeling and the recruitment of specific transcription factors at the RPL11 promoter awaits further analysis, potentially using single‐cell epigenomics. Finally, we must acknowledge that while the subcutaneous and biomimetic xenograft models used in this study effectively validated the core mechanism, they still cannot fully recapitulate the extremely complex stromal barrier and the complete host immune microenvironment of human PDAC. Therefore, we plan to introduce KPC conditional knockout mouse models and patient‐derived xenograft (PDX) models in follow‐up studies to conduct a deeper assessment of the long‐term anti‐tumor activity and immunomodulatory effects of this combination strategy in a more physiologically and clinically relevant host context.

## 4 Conclusion

In conclusion, this study systematically elucidates a novel resistance evolution axis in PDAC composed of “Lactate‐H3K18la‐RPL11.” Through an elegant mechanism of functional compartmentalization, this axis achieves a dual synergy of translational remodeling and DNA damage repair, constructing a robust chemoresistance defense. This work not only redefines the non‐canonical role of ribosomal proteins in chemoresistance but also provides a solid theoretical foundation and a tangible preclinical translational strategy for overcoming refractory PDAC by co‐targeting this metabolic‐epigenetic signaling axis.

## 5 Materials and Methods

### 5.1 Clinical Specimens and Ethical Statement

A retrospective cohort of 20 tumor specimens was obtained from patients with PDAC who received neoadjuvant chemotherapy (GEM combined with nab‐paclitaxel) at the Department of Pancreatobiliary Surgery, Sun Yat‐sen University Cancer Center (SYSUCC). Treatment response was evaluated according to the Response Evaluation Criteria in Solid Tumors (RECIST v1.1). Ten patients achieved a partial response (PR) and were categorized into the GEM‐sensitive group, while ten patients with stable disease (SD) were categorized into the GEM‐R group. All enrolled patients presented with borderline resectable or locally advanced PDAC and successfully underwent curative‐intent radical pancreatectomy (R0 or R1 margin) following 2–4 cycles of neoadjuvant therapy. This study was approved by the Institutional Review Board and Ethics Committee of SYSUCC (Approval No. SL‐B2025‐868‐01). Written informed consent was obtained from all participants prior to specimen collection.

### 5.2 Cell Lines and Generation of Resistant Models

Human (BxPC‐3, PANC‐1) and murine (Panc02) PDAC cell lines were obtained from the Cell Bank of the Chinese Academy of Sciences and cultured in DMEM with 10% FBS. GEM‐R sublines were established by continuous exposure to escalating GEM concentrations (starting at 5 µm) over 2–3 months. Resistance stability was validated by periodic IC_50_ determination. Cell viability and IC_50_ were assessed using the Cell Counting Kit‐8(CCK‐8) (#B34304, Bimake, Houston, TX, USA), and apoptosis was quantified via Annexin V‐FITC/7‐AAD flow cytometry (#40311ES60, Yeasen, Shanghai, China). Detailed protocols are provided in Supplementary Materials and Methods.

### 5.3 In Vivo Xenograft Models and Combination Therapy Assessment

All animal procedures were conducted in strict accordance with the Guidelines for the Welfare and Ethics of Laboratory Animals and were formally approved by the Institutional Animal Care and Use Committee (IACUC) of Xinxiang Medical University (Approval No. XYLL‐20250130). Male BALB/c nude mice (6–8 weeks old) were purchased from GemPharmatech (Jiangsu, China) and maintained in a specific‐pathogen‐free (SPF) facility.

#### 5.3.1 Biomimetic PDAC Xenograft Model

To faithfully recapitulate the dense, desmoplastic, and lactate‐rich microenvironment characteristic of human PDAC, BxPC‐3 cells (5×10^6^) stably engineered for target gene knockdown or overexpression were resuspended in 100 µL of a 1:1 (v/v) mixture of sterile PBS and high‐concentration Matrigel (Corning, GFR). The suspension was implanted subcutaneously into the right flank of each mouse. For initial mechanistic validations, a syngeneic model was established using murine Panc02 cells (2×10^6^) injected in the absence of Matrigel.

#### 5.3.2 Pharmacodynamics and Efficacy Evaluation

When tumor volumes reached approximately 50–80 mm^3^, mice were randomized into treatment cohorts. GEM (50 mg/kg, i.p., twice weekly) served as the foundational chemotherapy. Combination regimens included LDHi/Oxamate (750 mg/kg, i.p., daily), CX‐5461 (50 mg/kg, p.o., thrice weekly), or Olaparib (50 mg/kg, p.o., daily). Control groups received equivalent volumes of vehicle (saline or DMSO). Treatment continued for 3–4 weeks, during which tumor volume and body weight were measured weekly.

At the experimental endpoint, mice were euthanized, and tumors were excised and weighed. Blood samples were collected for biochemical analysis of liver and kidney function (ALT, AST, BUN, and CREA). Major organs (heart, liver, spleen, lungs, and kidneys) were harvested, fixed in 4% paraformaldehyd, and processed for hematoxylin and eosin (H&E) staining to assess histopathological changes.

### 5.4 Molecular and Biochemical Analyses

Standard protocols were used for Western blotting (WB), IF, and IHC. Primary antibodies included RPL11 (Proteintech), PARP14 (GeneTex), γ‐H2AX (ABclonal), and H3K18la (PTM Biolabs). Protein interactions were investigated via Co‐IP using Protein A/G beads, and chromatin occupancy by ChIP utilizing the SimpleChIP Plus Kit (CST). Protein identification was performed by LC‐MS/MS (BGI, Shenzhen) after silver staining. Gene expression was quantified by RT‐qPCR using the 2^−ΔΔCt^ method. Detailed protocols, antibody information, and primer sequences are provided in Supporting Information Materials and Methods and Table S1.

### 5.5 Subcellular Fractionation

Nuclear and cytoplasmic components were separated using the NE‐PER Nuclear and Cytoplasmic Extraction Reagents (#78835, Thermo Fisher Scientific) according to the manufacturer's optimized protocol. Cell pellets were lysed in cytoplasmic extraction reagents A and B supplemented with protease and RNase inhibitors. Following centrifugation at 12,000×g for 5 min at 4°C, the supernatant (cytoplasmic fraction) was collected. The remaining pellet was resuspended in nuclear extraction buffer and incubated on ice for 40 min, followed by centrifugation at 12 000×g for 10 min at 4°C to collect the nuclear fraction. Fractionation efficiency and purity were strictly cross‐verified by Western blotting using Histone H3 (nuclear marker) and GAPDH or α*α*‐Tubulin (cytoplasmic markers).

### 5.6 Lactate Level Measurement

Intracellular L‐lactate concentrations were quantified using an L‐Lactate Assay Kit (WST‐8 method; #S0208S, Beyotime) following the manufacturer's instructions. Absorbance was measured at 450 nm to calculate lactate concentrations.

### 5.7 Ribosome Analysis (SUnSET, Ribo‐Seq, and Polysome Profiling)

#### 5.7.1 Surface Sensing of Translation (SUnSET)

Global protein synthesis flux was assessed by measuring the incorporation of puromycin into nascent polypeptide chains. Cells were pulsed with 10 µg/mL puromycin (Sigma–Aldrich) for 30 min prior to harvest. Puromycin‐labeled proteins were then detected by Western blotting using a high‐specificity anti‐puromycin monoclonal antibody (Millipore, #MABE343).

#### 5.7.2 Ribo‐Seq and Polysome Profiling

Translation was arrested by pre‐treating cells with 100 µg/mL CHX for 5 min. For **Ribo‐seq**, samples were processed by Novogene (Beijing, China) for RNase I digestion, library construction, and high‐depth sequencing. For **Polysome profiling**, cell lysates were layered onto a 5%–50% (w/v) linear sucrose gradient and subjected to ultracentrifugation at 222 228 × g (TH‐641 rotor) for 2 h at 4°C. Fractions were continuously collected using a Piston Gradient Fractionator (BioComp Instruments), with simultaneous monitoring of ultraviolet absorbance at 260 nm to resolve 40S/60S subunits, monosomes, and polysome distributions.

### 5.8 DNA Damage Assessment (Comet Assay)

Single‐cell DNA fragmentation was quantified using a high‐throughput CometAssay system (Beyotime, #C2041M). Briefly, cells were harvested and resuspended in low‐melting‐point agarose, followed by rapid immobilization onto specialized glass slides. After overnight incubation in pre‐cooled lysis buffer at 4°C, two types of electrophoretic analyses were performed: Alkaline Comet Assay (detecting both single‐ and double‐strand breaks (DSBs): Slides were equilibrated in an alkaline unwinding buffer (pH > 13) for 30 min and subjected to electrophoresis at 25 V for 25 min. Neutral Comet Assay (specifically detecting DSBs): Slides were subjected to electrophoresis in TBE buffer (pH 7.5) for 45 min without the unwinding step. Post‐electrophoresis, slides were neutralized, stained with SYBR Green I, and visualized via confocal microscopy. At least 100 cells per group were randomly captured and analyzed using the Open Comet plugin in ImageJ. The percentage of DNA in the tail (% Tail DNA) and the Olive tail moment were quantified in a blinded manner to ensure objectivity.

### 5.9 Co‐Immunoprecipitation (Co‐IP) and IP‐MS Proteomics

Total protein was extracted using a non‐denaturing NP‐40 lysis buffer. Crucially, the lysates were supplemented with 25 U/mL universal nuclease (Benzonase) and incubated for 30 min at 4°C to achieve complete DNA/RNA degradation, thereby rigorously excluding false‐positive interactions mediated by nucleic acid bridging. Lysates (1 mg) were incubated with 2 µg of specific antibodies (e.g., anti‐H3K18la or anti‐RPL11) or isotype‐matched IgG at 4°C overnight. Immune complexes were captured using pre‐washed Pierce Protein A/G Magnetic Beads (Thermo Fisher, #88803) for 2 h. After five stringent washes with buffer containing 0.1% NP‐40, proteins were eluted in 1×SDS loading buffer containing β‐mercaptoethanol by boiling for 10 min.

For **Mass Spectrometry (IP‐MS)**, eluates were resolved by SDS‐PAGE and visualized with the Pierce Silver Stain Kit (e.g., Pierce, #24600). Differential bands of interest were excised and subjected to in‐gel tryptic digestion followed by high‐resolution liquid chromatography‐tandem mass spectrometry (LC‐MS/MS) analysis (BGI, Shenzhen) to identify core hub proteins interacting with H3K18la.

### 5.10 Chromatin Immunoprecipitation (ChIP‐qPCR)

ChIP assays were performed using the SimpleChIP Plus Enzymatic Chromatin IP Kit (Cell Signaling Technology, #9005). Cells were cross‐linked in vivo with 1% formaldehyde for 10 min. After nuclear isolation, chromatin was enzymatically digested into fragments (average size 200–500 bp) using Micrococcal Nuclease, supplemented with mild sonication. Immunoprecipitation was conducted using anti‐H3K18la or normal IgG. Following stringent high‐salt washes, cross‐link reversal, and Proteinase K digestion, the purified DNA was recovered. Target gene enrichment was quantified by qPCR using specific primers, with results expressed as a percentage of the total input (% Input). Primer sequences for ChIP‐qPCR are listed in Table S1 C.

### 5.11 Bioinformatics Analysis

#### 5.11.1 Transcriptome Sequencing

Total RNA sequencing was performed by BGI (Shenzhen, China) on the Illumina platform. Differentially expressed genes were identified using the DESeq2 R package, with thresholds set at Q‐value < 0.05 and ∣log2FoldChange∣>1. Pathway enrichment analyses were conducted using GO, Kyoto Encyclopedia of Genes and Genomes (KEGG), and Reactome databases.

#### 5.11.2 Clinical Database Analysis

Multi‐omic PDAC data from TCGA‐PAAD and GEO datasets (GSE15471, GSE16515, GSE62452) were normalized using log2(TPM+1) transformation. The GEPIA2 server and R *survival* package were utilized for differential expression analysis and Kaplan‐Meier survival curve plotting. PPI networks were constructed and visualized based on the STRING database (v11.5).

### 5.12 Statistical Analysis

All quantitative data were derived from at least three independent biological replicates and are presented as Mean ± Standard Deviation (SD). Statistical analyses were performed using GraphPad Prism v9.0. Prior to parametric testing, data were subjected to the Shapiro–Wilk test for normality and the Brown‐Forsythe test for homogeneity of variance. Comparisons between two groups were conducted using two‐tailed, unpaired Student's t‐tests. For multi‐group comparisons, one‐way or two‐way analysis of variance (ANOVA) followed by Tukey's or Sidak's post‐hoc tests were employed. Survival rates were compared using the Log‐rank (Mantel‐Cox) test. A *p*‐value < 0.05 was considered statistically significant (^*^ *p*<0.05, ^**^ *p*<0.01, ^***^ *p*<0.001; n.s., not significant).

## Author Contributions

L.J.Z, J.Z.C. and W.Y.L, contributed equally to this work. L.J.Z, R.Z., A.G.Y., J.J.L. and H.L.Y performed conceptualization; L.J.Z., J.Z.C., W.Y.L., X.Y.L., H.L.Z., X.F.Z and S.Y.S, performed data curation; L.J.Z, J.Z.C. and W.Y.L. performed formal Analysis; L.J.Z, and R.Z. performed Funding acquisition; L.J.Z, J.Z.C., W.Y.L, X.Y.L., H.L.Z, X.F.Z and S.Y.S performed investigation; L.J.Z, J.Z.C., and R.Z. performed methodology; L.J.Z, R.Z., A.G.Y., J.J.L. and H.L.Y performed project administration; L.J.Z, J.Z.C., and R.Z. acquired resources; L.J.Z, J.Z.C., W.Y.L and X.Y.L. acquired software; L.J.Z, R.Z., A.G.Y., J.J.L. and H.L.Y performed supervision; L.J.Z, J.Z.C., W.Y.L, X.Y.L., H.L.Z., X.F.Z. and S.Y.S performed validation; L.J.Z, J.Z.C., W.Y.L, X.Y.L., H.L.Z. and X.F.Z performed visualization; L.J.Z, J.Z.C., W.Y.L, and R.Z. wrote the original draft; L.J.Z, R.Z., A.G.Y., J.J.L. and H.L.Y. reviewed and edited the final manuscript; All authors read and approved the manuscript.

## Conflicts of Interest

The authors declare no conflicts of interest.

## Supporting information




**Supporting File 1**: advs78160‐sup‐0001‐SuppMat.docx.

**Supporting File 2**: advs78160‐sup‐0002‐TableS2.xlsx.

**Supporting File 3**: advs78160‐sup‐0003‐TableS3.xlsx.

**Supporting File 4**: advs78160‐sup‐0004‐TableS4.xlsx.

**Supporting File 5**: advs78160‐sup‐0005‐TableS5.xlsx.

**Supporting file 6**: advs78160‐sup‐0006‐TableS6.xlsx.

**Supporting file 7**: advs78160‐sup‐0007‐TableS7.xlsx.

## Data Availability

All data needed to evaluate and reproduce the results are present in the paper and/or the Supporting Information. The other data that support the findings of this study are available from the corresponding author upon reasonable request.
