# Supplementary material for: Lactate‐H3K18la‐RPL11 Axis Drives Gemcitabine Resistance in Pancreatic Cancer by Integrating Protein Synthesis and DNA Damage Repair
Source: Adv Sci (Weinh). 2026 Oct 8:e78160. Online ahead of print. doi: 10.1002/advs.78160 (PMC13647039; doi:10.1002/advs.78160)
Supplement: Supplementary file 1 [file ADVS-9999-e78160-s001.docx]

**Lactate-H3K18la-RPL11 Axis Drives Gemcitabine Resistance in Pancreatic Cancer by Integrating Protein Synthesis and DNA Damage Repair**

Lijun Zhao ^1,2, #^, Jianzhong Cao^3, #^, Wenyue Liu^2, #^, Xuyun Li^2^, Haolin Zhang^1^, Xiaofang Zhang^1^, Shiyang Shi^1^, Huilong Yin^2, *^, Junjie Li ^4, *^, Angang Yang ^1,2, *^ and Rui Zhang^1,*^

**Supporting Information**

**Contents**

1. Supplementary Figure and Legends: ………………………………2-15

2. Supplementary Materials and Methods: …………………………. 15-18

3. Supplementary Tables: ……………………………………………. 18-21

**1.** **Supplementary Figures and Legends**

**FIGURE S1. Lactate accumulation drives acquired gemcitabine (GEM) resistance and malignant phenotypes via upregulating H3K18la modification.** (A) Western blot analysis of pan-lysine lactylation (Pan-Kla) and specific histone H3 lactylation sites (H3K23la, H3K9la, H3K14la, H3K18la) in normal pancreatic tissues (N1-N2) and tumor tissues from GEM-sensitive (S1-S4) and GEM-resistant (R1-R4) PDAC patients. Total H3 served as the loading control. (B, C) Dose-response cell viability curves and corresponding IC50 values of (B) parental Panc02 and (C) acquired resistant Panc02-R cells treated with varied concentrations of GEM. (D) Quantitative analysis of basal intracellular lactate levels in Panc02 and Panc02-R cells (n = 3). (E) Western blot analysis of dynamic changes in Pan-Kla and H3K18la levels in Panc02-R cells after continuous treatment with 5 μM GEM for the indicated times (days 7 to 28). (F, G) Following treatment of parental Panc02 cells with exogenous sodium L-lactate (0, 10, 50 mM): (F) Western blot validation of Pan-Kla and H3K18la expression, and (G) cell viability curves upon combined GEM treatment. (H, I) Following treatment of Panc02-R cells with an LDHA inhibitor (LDHi; 0, 10, 50 μM): (H) Western blot validation of decreased Pan-Kla and H3K18la levels, and (I) cell viability curves upon combined GEM treatment. (J, K) Upon LDHA knockdown (LDHA-KD) in Panc02-R cells: (J) Western blot analysis of Pan-Kla and H3K18la levels, and (K) viability curves showing restored sensitivity to GEM. (L, M) After supplementation with lactate, acetate, or pyruvate in LDHA-knockdown Panc02-R cells: (L) Western blot analysis of Pan-Kla and H3K18la levels, and (M) GEM dose-response cell viability curves. (N, O) Effects of exogenous lactate treatment on Panc02 cell phenotypes: (N) CCK-8 proliferation curves and (O) flow cytometry analysis of apoptosis. (P, Q) Effects of LDHi treatment on Panc02-R cell phenotypes: (P) CCK-8 proliferation curves and (Q) flow cytometry analysis of apoptosis. Data are presented as mean ± SD (n = 3 independent experiments). Statistical significance was assessed by two-tailed Student's t-test. **p < 0.05, **p < 0.01, ***p < 0.001.*

**FIGURE S2. The "lactate-H3K18la" axis reshapes cellular translation flux to mediate chemoresistance by upregulating and physically interacting with RPL11.** (A) Silver staining analysis of H3K18la immunoprecipitation (IP) complexes in BxPC-3-R and Panc02-R cells. Black arrows indicate potential differentially bound protein bands (including RPL11). (B) Clustered heatmap of differentially expressed genes (DEGs) regulated by H3K18la in PANC-1 cells, based on a public transcriptomic dataset (GSE290845). (C) MCODE cluster analysis based on the protein-protein interaction (PPI) network, highlighting core gene modules enriched in ribosome biogenesis and translation-related biological processes. (D, E) RT-qPCR (top) and Western blot (bottom) analyses of RPL11 expression levels in (D) Panc02 and (E) PANC-1 cells treated with exogenous lactate. (F) Western blot analysis of Pan-Kla, H3K18la, and RPL11 protein levels in Panc02-R cells upon RPL11 knockdown under basal conditions and lactate stimulation. (G) CCK-8 assay evaluating the effect of RPL11 knockdown on GEM resistance in Panc02-R cells under basal conditions and lactate stimulation. (H, I) Flow cytometry analysis of apoptosis (H) and corresponding quantitative statistics (I) in Panc02-R cells upon RPL11 knockdown under basal conditions and lactate stimulation. (J) Immunofluorescence (IF) staining showing the expression abundance and subcellular localization of RPL11 (red) in parental Panc02 and resistant Panc02-R cells under basal conditions and lactate stimulation. Nuclei were counterstained with DAPI (blue). (K) Co-immunoprecipitation (Co-IP) assay confirming the direct interaction between endogenous H3K18la and RPL11 proteins in Panc02-R and BxPC-3-R cells. (L) RT-qPCR analysis of dynamic effects of lactate stimulation (top) or LDHi treatment (bottom) on the expression levels of RPL11 and pre-ribosomal RNA (18S/28S rRNA). (M) SUnSET assay assessing the effects of lactate stimulation (left) or LDHi treatment (right) on global protein synthesis rates in Panc02-R cells. GAPDH served as the internal control. Data are presented as mean ± SD (n = 3) and statistically analyzed using two-tailed Student's t-test. **p < 0.05, **p < 0.01, ***p < 0.001.*

**FIGURE S3. H3K18la promotes RPL11 transcriptional expression by directly binding to its promoter region.** (A, C) Dual-luciferase reporter assays detecting the effects of wild-type H3 or the non-lactylated mutant H3K18R on RPL11 promoter activity in (A) Panc02-R or (C) BxPC-3-R cells. (B, D) IF staining of RPL11 (red) expression in (B) Panc02-R or (D) BxPC-3-R cells. Scale bars: 20 μm (1200×). (E, F) Chromatin immunoprecipitation-quantitative PCR (ChIP-qPCR) analysis of H3K18la enrichment at different regions (P1-P3) of the RPL11 promoter in (E) Panc02-R or (F) BxPC-3-R cells, assessing enrichment changes following treatment with a P300 inhibitor (P300i) or LDHi. (G, H) RT-qPCR analysis of RPL11 mRNA expression levels in (G) Panc02-R or (H) BxPC-3-R cells treated with P300i or LDHi. (I, J) Western blot analysis of Pan-Kla, H3K18la, and RPL11 protein levels in (I) Panc02-R or (J) BxPC-3-R cells after P300i or LDHi treatment. Actin served as the loading control. Data are expressed as mean ± SD from 3 independent replicates (n = 3). Statistical analysis was performed using Student's t-test. **p < 0.05, **p < 0.01, ***p < 0.001.*

**FIGURE S4. RPL11 drives GEM resistance and regulates tumor growth in vitro and in vivo.** (A, B, K, L) RT-qPCR (top) and Western blot (bottom) validation of stable RPL11 overexpression (OE) and knockdown (sh-RPL11) efficiencies in PANC-1 (A, B) and Panc02 (K, L) cells. (C, D, M, N) GEM dose-response curves and IC50 values evaluating the impact of RPL11 modulation on chemoresistance in PANC-1 (C, D) and Panc02 (M, N) cells. (E, H, O, R) CCK-8 assays evaluating the effects of RPL11 OE or knockdown on the growth potential of PANC-1 (E, H) and Panc02 (O, R) cells. (F-G, I-J, P-Q, S-T) Flow cytometry analysis (Annexin V-FITC/7-AAD staining) assessing the effects of RPL11 expression levels on apoptosis in PANC-1 (F-J) and Panc02 (P-T) cells, presenting representative scatter plots and quantitative analysis of late apoptosis rates. (U, V) Subcutaneous Panc02 xenograft models (n = 5-6/group) assessing the effects of RPL11 OE (U) or knockdown (V) on in vivo tumor growth. Representative gross tumor images, tumor weights, and volume growth curves are shown. Data are presented as mean ± SD. Statistical significance was analyzed by two-tailed Student's t-test. **p < 0.05, **p < 0.01, ***p < 0.001,* ns: not significant.

**FIGURE S5. RPL11 confers cross-resistance to broad-spectrum DNA damage-inducing chemotherapeutic drugs in PDAC cells.** (A, E, G, I) Dose-response cell viability curves and IC50 values showing changes in sensitivity to (A) oxaliplatin, (E) 5-fluorouracil (5-FU), (G) irinotecan, and (I) paclitaxel following RPL11 overexpression (pCDH vs. RPL11) in parental PancO2 cells.(B, F, H, J) Dose-response cell viability curves and IC50 values showing changes in sensitivity to (B) oxaliplatin, (F) 5-FU, (H) irinotecan, and (J) paclitaxel following RPL11 knockdown (pLKO.1 vs. sh-RPL11) in resistant PancO2-R cells.(C, D) IF staining evaluating the effects of RPL11 overexpression in parental PancO2 cells (C) and RPL11 knockdown in resistant PancO2-R cells (D) on the foci formation of the DNA damage marker γH2AX (red) under oxaliplatin treatment. Nuclei were counterstained with DAPI (blue).

**FIGURE S6. EEF1A1 promotes gemcitabine resistance and malignant progression in PDAC cells.** (A, B, K, L) RT-qPCR (top) and Western blot (bottom) validation of stable overexpression (OE) and knockdown (sh-EEF1A1 #1, #2, #3) efficiencies of EEF1A1 in BxPC-3 (A, B) and Panc02 (K, L) cells.(C, D, M, N) GEM dose-response curves and IC50 values evaluating the regulatory role of EEF1A1 in the chemosensitivity of BxPC-3 (C, D) and Panc02 (M, N) cells.(E, H, O, R) CCK-8 proliferation kinetics curves showing the impact of EEF1A1 expression levels on the growth potential of BxPC-3 (E, H) and Panc02 (O, R) cells.(F-G, I-J, P-Q, S-T) Flow cytometry analysis (Annexin V-FITC/7-AAD) evaluating the effects of EEF1A1 modulation on apoptosis in BxPC-3 (F-J) and Panc02 (P-T) cells. Representative scatter plots and quantitative analysis of late apoptosis rates are shown. Data are presented as mean ± SD (n = 3). Statistical significance was analyzed by two-tailed Student's t-test. **p < 0.05, **p < 0.01, ***p < 0.001,* ns: not significant.

**FIGURE S7. The oncogenic role of RPL11 in gemcitabine resistance strictly relies on its downstream effector EEF1A1.**(A, B) Western blot validation of the construction efficiencies of the rescue models in (A) BxPC-3 and (B) Panc02 cells, showing the changes in the expression levels of both proteins upon simultaneous intervention of EEF1A1 in the background of RPL11 overexpression or knockdown.(C) SUnSET assay (puromycin labeling) detecting the effects of EEF1A1 intervention on the de novo protein synthesis rates in the background of RPL11 overexpression or knockdown.(D, F) GEM dose-response curves and IC50 values assessing the restorative effects of EEF1A1 intervention on chemosensitivity in the background of RPL11 (D) overexpression or (F) knockdown.(E, G) Proliferation kinetics curves showing the impact of EEF1A1 intervention on cell growth capacity in the background of RPL11 (E) overexpression or (G) knockdown.(H, I) Flow cytometry analysis and quantitative statistics evaluating the effects of EEF1A1 intervention on late apoptosis rates in the background of RPL11 (H) overexpression or (I) knockdown. Statistical significance was analyzed by two-tailed Student's t-test. **p < 0.05, **p < 0.01, ***p < 0.001.*

**FIGURE S8. H3K18la-induced RPL11 translocates into the nucleus to directly transcriptionally activate PARP14 and drive DNA damage repair.**(A, B) RT-qPCR analysis of mRNA expression changes of RPL11 and its downstream target PARP14 following RPL11 overexpression in parental PANC-1 cells (A) and RPL11 knockdown in resistant PANC-1-R cells (B).(C, D) Chromatin immunoprecipitation-quantitative PCR (ChIP-qPCR) evaluating the enrichment levels of RPL11 at different predicted regions (P-1 to P-4) of the PARP14 promoter in (C) Panc02-R and (D) PANC-1-R cells, confirming their direct binding at the P-3 region. (E, F) Verification of Importin-β-mediated nuclear translocation mechanism: Following treatment with the nuclear transport inhibitor Importazole, (E) immunofluorescence staining (red for RPL11) and (F) nuclear-cytoplasmic fractionation Western blot assays (H3 and GAPDH as nuclear/cytoplasmic internal controls, respectively) confirmed that Importazole effectively blocked the nuclear accumulation of RPL11 under basal conditions and lactate stimulation.(G) RT-qPCR analysis of the combined effects of lactate stimulation and Importazole-mediated nuclear translocation blockade on the transcriptional levels of RPL11 and its downstream target PARP14.(H,I) Dose-response cell viability curves showing the viability changes of Panc02 and Panc02-R cells treated with lactate and/or Importazole in combination with gemcitabine (GEM), confirming that blocking RPL11 nuclear translocation effectively abrogates the lactate-mediated chemoprotective effects. (J, K) H3K18la-targeted ChIP-qPCR analysis: assessing the dynamic enrichment of H3K18la at the (J) PARP14 promoter (P-1 to P-4) and (K) RPL11 promoter (P-1 to P-3) regions under basal conditions, exogenous lactate stimulation, and LDHA inhibitor (LDHi) treatment. Data are expressed as mean ± SD. Statistical differences were assessed by Student's t-test. ****p < 0.001.*

**FIGURE S9. PARP14 is significantly overexpressed in PDAC and is closely associated with poor prognosis and malignant phenotypes.**(A-D) Differential expression analysis of PARP14 between pancreatic ductal adenocarcinoma (PDAC) tissues and adjacent normal tissues based on (A) TCGA and GEO datasets (B) GSE16515, (C) GSE15471, and (D) GSE62452.(E) Correlation analysis between PARP14 expression levels and tumor pathological grades based on the GSE62452 dataset.(F, G) Kaplan-Meier survival curves evaluating the association between PARP14 expression abundance and overall survival (OS) in the (F) TCGA and (G) GSE62452 patient cohorts.(H, I) RT-qPCR (top) and Western blot (bottom) validation of the construction efficiencies of PARP14 (H) stable overexpression and (I) knockdown using three independent shRNAs in BxPC-3 cells.(J, N) GEM dose-response curves and IC50 values evaluating the regulatory role of PARP14 (J) overexpression or (N) knockdown on chemoresistance in PDAC cells.(K, O) CCK-8 proliferation kinetics curves evaluating the impact of PARP14 alterations on cell growth capacity.(L-M, P-Q) Flow cytometry (Annexin V-FITC/7-AAD) detection and quantitative statistics evaluating the effects of PARP14 expression alterations on apoptosis.(R, S) Subcutaneous xenograft models utilizing BxPC-3 and BxPC-3-R cells to assess the effects of PARP14 (R) overexpression or (S) knockdown on in vivo tumor growth. Representative gross tumor images, volume growth curves, and terminal tumor weights are shown. Data are presented as mean ± SD (in vitro experiments n = 3; in vivo experiments n = 6 mice/group). Comparisons between two groups were analyzed by two-tailed Student's t-test; survival analysis was performed using the Log-rank test. **p < 0.05, **p < 0.01, ***p < 0.001.*

**FIGURE S10. PARP14 promotes gemcitabine resistance and malignant progression in PANC-1 and Panc02 cells.**(A, D, G, J) RT-qPCR (top) and Western blot (bottom) validation of stable PARP14 overexpression (OE) and knockdown (sh-PARP14 #1, #2, #3) efficiencies in (A, D) PANC-1 and (G, J) Panc02 cells.(B, E, H, K) GEM dose-response curves assessed by CCK-8 assay, with calculated Log IC50 and IC50 values.(C, F, I, L) CCK-8 proliferation kinetics curves (96 hours) evaluating the impact of PARP14 on cell growth potential.(M-P) Representative flow cytometry scatter plots showing the effects of PARP14 on cell survival status, with quantitative statistics of late apoptosis rates shown on the right. Data are presented as mean ± SD (n = 3 independent experiments). Statistical significance was assessed by two-tailed Student's t-test. **p < 0.05, **p < 0.01, ***p < 0.001.*

**FIGURE S11. Molecular mechanisms and rescue validation of the RPL11-PARP14 signaling axis in driving gemcitabine resistance and DNA damage repair in PDAC cells.** (A) RNA immunoprecipitation-quantitative PCR (RIP-qPCR) analysis confirming the direct binding and enrichment between RPL11 protein and PARP14 mRNA in resistant cell lines (Panc02-R and PANC-1-R). (B, C) Actinomycin D transcription blockade assay evaluating the half-life of PARP14 mRNA. Dynamic degradation curves of PARP14 mRNA over time are shown for (B) parental cells (Panc02 and BxPC-3) with RPL11 overexpression (pCDH vs. RPL11) and (C) resistant cells (Panc02-R and BxPC-3-R) with RPL11 knockdown (pLKO.1 vs. sh-RPL11). (D, E) Validation of the construction efficiency of functional rescue cell models. RT-qPCR (top) and Western blot (bottom) showing the expression levels in (D) PANC-1 cells with RPL11 overexpression alone or combined with PARP14 intervention (overexpression/knockdown), and (E) PANC-1-R cells with RPL11 knockdown alone or combined with PARP14 intervention (restoration/knockdown). Actin served as the loading control.(F-H) Regulation of malignant phenotypes in parental PANC-1 cells by the RPL11-PARP14 axis: Evaluation of (F) dose-response viability curves and IC50 values under gemcitabine (GEM) treatment, (G) CCK-8 proliferation kinetics curves, and (H) apoptosis rates with quantitative analysis detected by flow cytometry (Annexin V-FITC/PI) among different transfection groups.(I-K) Regulation of malignant phenotypes in resistant PANC-1-R cells by the RPL11-PARP14 axis: Evaluation of (I) GEM dose-response viability curves and IC50 values, (J) CCK-8 proliferation kinetics curves, and (K) flow cytometric analysis of apoptosis rates and quantification among different transfection groups.(L-O) Assessment of the impact of targeting the RPL11-PARP14 axis on cellular DNA damage repair capacity. Comet assays (displaying DNA tailing) combined with γH2AX (red) immunofluorescence staining (indicating DNA damage foci formation) are shown for the rescue models in (L, M) PANC-1 cells and (N, O) PANC-1-R cells. Nuclei were counterstained with DAPI (blue). Scale bars: 20 μm. Data are presented as mean ± SD (n = 3 independent experiments). Statistical significance was assessed by two-tailed Student's t-test. **p < 0.05, **p < 0.01, ***p < 0.001.*

**FIGURE S12. RPL11 positively regulates PARP14 to enhance DNA damage repair and induce gemcitabine resistance.** (A) RT-qPCR detection of PARP14 mRNA levels in Panc02 cells following RPL11 overexpression (top) or knockdown (bottom). (B) Dynamic expression profile of PARP14 mRNA in BxPC-3 cells during continuous GEM treatment (5 μM, day 0 to 28). (C, D) Assessment of the expression abundance, subcellular localization, and co-localization of RPL11 (red) and PARP14 (green) in Panc02 cells under lactate stimulation or GEM resistance conditions via (C) nucleocytoplasmic fractionation Western blot and (D) immunofluorescence co-staining. Histone H3 and GAPDH served as nuclear and cytoplasmic internal controls, respectively. Scale bars: 20 μm. (E, F) RT-qPCR (top) and Western blot (bottom) validation of rescue model efficiencies with simultaneous PARP14 intervention in the background of (E) RPL11 overexpression or (F) RPL11 knockdown. (G-L) Evaluation of the RPL11-PARP14 axis on GEM resistance, proliferation, and apoptosis. Shown are (G, J) GEM dose-response curves, (H, K) proliferation kinetics curves, and (I, L) flow cytometry (Annexin V-FITC/7-AAD) apoptosis analyses in the rescue model groups. (M-P) Evaluation of DNA damage and repair capabilities in the aforementioned rescue models using (M, O) Comet assays to assess overall DNA integrity (scale bars: 20 μm) and (N, P) γH2AX immunofluorescence staining to quantify DNA double-strand break (DSB) foci formation (scale bars: 10 μm). Data are mean ± SD (n = 3). Statistical significance was assessed by two-tailed Student's t-test. **p < 0.05, **p < 0.01, ***p < 0.001, ****p < 0.000*1.

**FIGURE S13. PARP inhibitors combined with gemcitabine synergistically reverse chemoresistance in PDAC cells in vitro and in vivo.** (A, C) Dose-response curves of resistant cell lines (A) BxPC-3-R and (C) Panc02-R treated with monotherapy or combined regimens. Embedded tables list the corresponding Log IC50 and IC50 values, demonstrating significant synergistic sensitization (GEM+PARPi).(B, D) Proliferation kinetics curves showing that the combined regimen significantly inhibits the in vitro growth capacity of (B) BxPC-3-R and (D) Panc02-R cells compared with monotherapy.(E, F) Flow cytometry (Annexin V-FITC/PI) detection and quantitative statistics of the effects of indicated treatments on the late apoptosis rates of (E) BxPC-3-R and (F) Panc02-R cells.(G, H) Subcutaneous xenograft models evaluating the in vivo therapeutic efficacy of the combined regimen. Representative gross tumor images, volume growth curves, and terminal tumor weights are shown for the (G) BxPC-3-R (n = 5) and (H) Panc02-R (n = 4) groups. (I) Serum biochemical analysis of liver function (ALT, AST) and kidney function (UREA, CREA) markers from tumor-bearing mice in (G) to assess the systemic biosafety of the combined regimen. Data are presented as mean ± SD. **p < 0.05, **p < 0.01, ***p < 0.001,* ns: not significant.

**FIGURE S14. Synergistic co-inhibition of lactate metabolism and ribosome biogenesis enhances gemcitabine efficacy with high clinical relevance.** (A, B) Evaluation of the inhibitory effects of GEM, LDH inhibitor (LDHi), and Pol I inhibitor (CX5461) as monotherapies or in combination on resistant cells via (A) proliferation kinetics and (B) dose-response curves (with corresponding IC50 values shown in the table). (C-D) Flow cytometry (Annexin V-FITC/7-AAD) quantification of apoptosis proportions induced by the indicated treatments. (E-G) Panc02-R subcutaneous xenograft models (n = 3) assessing the in vivo tumor-suppressive efficacy of the combined regimen. Terminal tumor weights, volume growth curves, and gross tumor images are shown. (H, J) Representative H&E staining images of major organs, including the (H) liver and (J) kidney, from mice in the combination therapy group to evaluate potential histopathological toxicity. Scale bars: 50 μm (200×) and 20 μm (400×). (I, K) Serum biochemical analyses of (I) liver function (AST, ALT) and (K) kidney function (CREA, UREA) across groups to assess systemic biosafety. (L, M) Representative immunohistochemistry (IHC) images (L) and corresponding H-score quantification (M) illustrating the differential expression of RPL11 in clinical tissue samples from 10 GEM-sensitive and 10 GEM-resistant PDAC patients. Scale bars: 50 μm (200×, top row) and 20 μm (400×, bottom row). (N) Pearson correlation analysis revealing a significant positive correlation between H3K18la levels and RPL11 expression in PDAC clinical samples. Data are presented as mean ± SD. Statistical significance was assessed by one-way ANOVA or Student's t-test. Correlation was evaluated using the Pearson correlation coefficient (r). **p < 0.05, **p < 0.01, ***p < 0.001.*

**2. Supplementary Materials and Methods**

**2.1** **Cell Lines and Culture**

Human PDAC cell lines (BxPC-3 and PANC-1) and the murine PDAC cell line (Panc02) were purchased from the American Type Culture Collection (ATCC; Manassas, VA, USA) or the Cell Bank of the Chinese Academy of Sciences (Shanghai, China). Cells were cultured in high-glucose Dulbecco’s Modified Eagle Medium (DMEM; #12800082, Gibco, Grand Island, NY, USA) supplemented with 10% fetal bovine serum (FBS; Gibco), 100 U/mL penicillin, and 100 µg/mL streptomycin. All cells were maintained in a humidified incubator at 37°C with 5% CO₂ and were routinely tested for *Mycoplasma* contamination.

**2.2 Reagents and Antibodies**

Gemcitabine (GEM, #HY-17026), LDHA inhibitor (LDHi, #HY-W013032A), CX-5461 (#HY-13323), PARP inhibitor Olaparib (#HY-10162), Oxaliplatin (#HY-17371), 5-Fluorouracil (5-FU, #HY-90006), Irinotecan hydrochloride (Irinotecan, #HY-16562A), Paclitaxel (#HY-B0015), Importazole (#HY-101091), Sodium dichloroacetate (#HY-Y0445A), and Sodium Pyruvate Solution (100 mM, #HY-K3024) were all purchased from MedChemExpress (MCE; Monmouth Junction, NJ, USA). All compounds were dissolved in dimethyl sulfoxide (DMSO; #D8371, Solarbio, Beijing, China) to prepare stock solutions, which were subsequently diluted with culture medium to the desired working concentrations immediately prior to use.

**2.3 Plasmid Construction and Lentiviral Transduction**

Plasmids for the overexpression of wild-type H3 (H3-WT), the H3K18R mutant, and the RPL11 promoter-luciferase reporter were purchased from Yixue Biotechnology (Shanghai, China). Plasmids for the overexpression of RPL11 (human #P105164, mouse #P41997), EEF1A1 (human #P99374, mouse #P105430), and PARP14 (human #P101265, mouse #P59579), as well as the shRNA plasmid targeting LDHA (LDHA sh, #P98156), were purchased from Miaoling Plasmid Platform and verified by Sanger sequencing.

For gene knockdown, shRNA sequences targeting specific genes were designed using Oligoengine software and validated by BLAST for specificity before being cloned into the pLKO.1 vector. Primer for construct knockdown plasmids and qPCR are listed in Supplementary Table S1 A-B.

**2.4 Establishment of Acquired Gemcitabine-Resistant (GEM-R) Cell Lines**

Acquired GEM-R cell lines (Panc02-R, BxPC-3-R, and PANC-1-R) were established through a chronic, stepwise dose-escalation protocol. Briefly, parental cells in the exponential growth phase were continuously exposed to gemcitabine at an initial concentration of 5 µM (approximating the parental IC50*IC*50). The concentration was incrementally increased over a period of 2–3 months as the cells adapted. Following successful establishment, GEM-R cells were maintained in medium containing a maintenance dose of gemcitabine. The drug was withdrawn one week prior to all experimental assays. The stability of the resistance phenotype was strictly monitored by monthly IC50*IC*50 determination via CCK-8 assays.

**2.5 Lentivirus Production and Stable Cell Line Generation**

Lentiviral expression or shRNA plasmids were co-transfected with packaging plasmids psPAX2 and pMD2.G into 293T cells at a molar ratio of 4:3:1 using PEI transfection reagent (#23966-1, Polysciences, Warrington, PA, USA). Supernatants containing viral particles were collected at 48 and 72 h post-transfection and filtered through a 0.45 µm filter. Log-phase PDAC cells were infected with the viral supernatants in the presence of Polybrene (8 µg/mL, Sigma-Aldrich). After 48 h, cells were selected with puromycin (5 µg/mL, Sigma-Aldrich) for 1–2 weeks to generate stable cell lines. Transduction efficiency was confirmed by RT-qPCR and Western Blot.

**2.6 Cell Viability and Half-Maximal Inhibitory Concentration (IC50)**

Cell proliferation and drug sensitivity were quantified using the Cell Counting Kit-8 (CCK-8; #B34304, Bimake, Houston, TX, USA). For proliferation assays, cells were seeded at a density of 1×10^3^ cells/well in 96-well plates. At designated time points (24, 48, 72, and 96 h), 10 µL of CCK-8 reagent was added, followed by incubation at 37°C for 2 h in the dark. For IC50 determination, cells (1×10^4^ cells/well) were allowed to adhere for 8 h before the medium was replaced with fresh medium containing serial dilutions of gemcitabine (ranging from 0 to 320 µM). After 48 h of treatment, absorbance at 450 nm was measured using a Multiskan MK3 microplate reader (Thermo Fisher Scientific). IC50 values were calculated using a non-linear regression model in GraphPad Prism v9.0.

**2.7 Flow Cytometric Analysis of Apoptosis**

Drug-induced apoptosis was precisely quantified using the Annexin V-FITC/7-AAD Double Staining Apoptosis Detection Kit (#40311ES60, Yeasen, Shanghai, China) or the Annexin V-FITC Apoptosis Detection Kit (Beyotime, C1062S). After treatment, all floating and adherent cells were harvested, washed twice with cold PBS, and resuspended in 100 µL of 1×Binding Buffer. Cells were then incubated with 5 µL of Annexin V-FITC and 5 µL of 7-AAD for 15 min at room temperature in the dark. Data were acquired within 1 h using a CytoFLEX flow cytometer (Beckman Coulter, Brea, CA, USA).

Offline analysis was performed using FlowJo v10.8 software. The gating strategy involved excluding cell debris (FSC-A/SSC-A) and doublets (FSC-H/FSC-A) to ensure single-cell population analysis. Early apoptotic (Annexin V+/7-AAD-) and late apoptotic (Annexin V+/7-AAD+) populations were quantified. To ensure absolute accuracy and reproducibility, gating and statistical calculations were performed by two independent researchers in a blinded manner.

**2.8 RNA Extraction and Real-Time Quantitative PCR (RT-qPCR)**

Total RNA was extracted using TRIzol® Reagent (#15596018CN, Invitrogen, Carlsbad, CA, USA). RNA was reverse-transcribed into cDNA using the PrimeScript RT Reagent Kit (#RR036A, Takara, Shiga, Japan). qPCR was performed on a CFX96™ Real-Time PCR Detection System (Bio-Rad, Hercules, CA, USA) using BeyoFast SYBR Green qPCR Mix (#11184ES03, Yeasen). Relative gene expression was calculated using the 2^⁻ΔΔCt^ method with β-actin as the internal control. All primer sequences are listed in Supplementary Table S1 B.

**2.9 mRNA Stability (Actinomycin D) Assay**

For mRNA stability assays, cells were treated with the transcriptional inhibitor Actinomycin D (5 µg/mL). Cells were harvested at 0, 2, 4, 6, and 8 h post-treatment, and total RNA was extracted for RT-qPCR analysis to determine the half-life of target mRNAs.

**2.10 Western Blotting (WB)**

Total protein was extracted using RIPA lysis buffer (#R0020, Solarbio) supplemented with protease and phosphatase inhibitors (Roche, Basel, Switzerland). Protein concentration was determined using the BCA assay (#SF007, Saphire, Shanghai, China). Equal amounts of protein (20–30 µg) were separated by 10-12% SDS-PAGE and transferred to PVDF membranes (Millipore, Burlington, MA, USA). Membranes were blocked with 5% non-fat milk in TBST for 1 h at room temperature, followed by overnight incubation with primary antibodies (dilutions in **Supplementary Table S1 D**) at 4°C. After washing, membranes were incubated with HRP-conjugated secondary antibodies (1:5000, Proteintech) for 1 h. Bands were visualized using an ECL substrate (Millipore) on a ChemiDoc XRS+ System (Bio-Rad, Hercules, CA, USA).

**2.11 Immunohistochemistry (IHC) and Immunofluorescence (IF)**

**IHC:** Paraffin-embedded sections were deparaffinized, rehydrated, and subjected to antigen retrieval in citrate buffer (pH 6.0). Endogenous peroxidase activity was quenched with 3% H₂O₂, and sections were blocked with 5% BSA. Primary antibodies were applied overnight at 4°C, followed by HRP-conjugated secondary antibodies. Staining was visualized with DAB and counterstained with hematoxylin. Images were captured using a Pannoramic 250 FLASH slide scanner (3DHISTECH, Budapest, Hungary). A semi-quantitative H-score (= Σ (pi × i), where pi is the percentage of positive cells and i is the staining intensity) was calculated.

**IF:** Cells grown on coverslips or cryosections were fixed with 4% paraformaldehyde, permeabilized with 0.2% Triton X-100, and blocked with 5% BSA. Primary antibodies were incubated overnight at 4°C, followed by Alexa Fluor-conjugated secondary antibodies (1:500, Invitrogen). Nuclei were counterstained with DAPI (#P0131, Beyotime, Shanghai, China). Images were acquired using a Zeiss LSM880 confocal microscope (Carl Zeiss, Oberkochen, Germany).

### ****3. Supplementary Tables****

**Supplementary Table S1. List of primers sequences used in this research.**

**A. Primer for construct knockdown plasmids**

|  | Symbol | Sequence |
| --- | --- | --- |
| h-RPL11 | h- RPL11- sh1 | F:CCGGGCGGGAGTATGAGTTAAGAAACTCGAGTTTCTTAACTCATACTCCCGCTTTTTG |
|  |  | R:AATTCAAAAAGCGGGAGTATGAGTTAAGAAACTCGAGTTTCTTAACTCATACTCCCGC |
|  | h-RPL11- sh2 | F:CCGGCCGCAAACTCTGTCTCAACATCTCGAGATGTTGAGACAGAGTTTGCGGTTTTTG |
|  |  | R:AATTCAAAAACCGCAAACTCTGTCTCAACATCTCGAGATGTTGAGACAGAGTTTGCGG |
|  | h-RPL11- sh3 | F:CCGGCAACTTCTCAGATACTGGAAACTCGAGTTTCCAGTATCTGAGAAGTTGTTTTTG |
|  |  | R:AATTCAAAAACAACTTCTCAGATACTGGAAACTCGAGTTTCCAGTATCTGAGAA GTTG |
| M-RPL11 | M - RPL11- sh1 | F:CCGGCGGATACTGGAAACTTTGGTTCTCGAGAACCAAAGTTTCCAGTATCCGTTTTTG |
|  |  | R:AATTCAAAAACGGATACTGGAAACTTTGGTTCTCGAGAACCAAAGTTTCCAGTATCCG |
|  | M -RPL11- sh2 | F:CCGGCGGGAGTATGAGTTGCGGAAACTCGAGTTTCCGCAACTCATACTCCCGTTTTTG |
|  |  | R:AATTCAAAAACGGGAGTATGAGTTGCGGAAACTCGAGTTTCCGCAACTCATACTCCCG |
|  | M -RPL11- sh3 | F:CCGGCGGAGAAATGAGAAGATTGCTCTCGAGAGCAATCTTCTCATTTCTCCGTTTTTG |
|  |  | R:AATTCAAAAACGGAGAAATGAGAAGATTGCTCTCGAGAGCAATCTTCTCATTTCTCCG |
| h-EEF1A1 | h- EEF1A1- sh1 | F:CCGGCCTCTCCAGGATGTCTACAAACTCGAGTTTGTAGACATCCTGGAGAGGTTTTTG |
|  |  | R:AATTCAAAAACCTCTCCAGGATGTCTACAAACTCGAGTTTGTAGACATCCTGGAGAGG |
|  | h-EEF1A1- sh2 | F:CCGGCCAGAAGAGATATGAGGAAATCTCGAGATTTCCTCATATCTCTTCTGGTTTTTG |
|  |  | R:AATTCAAAAACCAGAAGAGATATGAGGAAATCTCGAGATTTCCTCATATCTCTT CTGG |
|  | h-EEF1A1- sh3 | F:CCGGCCCAGGACACAGAGACTTTATCTCGAGATAAAGTCTCTGTGTCCTGGGTTTTTG |
|  |  | R:AATTCAAAAACCCAGGACACAGAGACTTTATCTCGAGATAAAGTCTCTGTGTCCTGGG |
| M-EEF1A1 | M-EEF1A1-sh1 | F:CCGGGCGTGGTATCACTATTGACATCTCGAGATGTCAATAG TGATACCACGC TTTTTG |
|  |  | R:AATTCAAAAAGCGTGGTATCACTATTGACATCTCGAGATGTCAA TAGTGATACCACGC |
|  | M-EEF1A1-sh2 | F:CCGGCCAGTCAATGTAACAACTGAACTCGAGTTCAGTTGTT ACATTGACTGG TTTTTG |
|  |  | R:AATTCAAAAACCAGTCAATGTAACAACTGAACTCGAGTTCAGTT GTTACATTGACTGG |
|  | M-EEF1A1-sh3 | F:CCGGCGTTCTGGTAAGAAGCTGGAACTCGAGTTCCAGCTTC TTACCAGAAC G TTTTTG |
|  |  | R:AATTCAAAAACGTTCTGGTAAGAAGCTGGAACTCGAGTTCCAGC TTCTTACCAG AACG |
| h-PARP14 | h- PARP14- sh1 | F:CCGGGCACCATTTGAAGAGTCACTACTCGAGTAGTGACTCT TCAAATGGTGCTTTTTG |
|  |  | R:AATTCAAAAAGCACCATTTGAAGAGTCACTACTCGAGTAGTGAC TCTTCAAATG GTGC |
|  | h-PARP14- sh2 | F:CCGGGATTGAGTTTGATACACTTAACTCGAGTTAAGTGTAT CAAACTCAATCTTTTTG |
|  |  | R:AATTCAAAAAGATTGAGTTTGATACACTTAACTCGAGTTAAGTG TATCAAACTCAATC |
|  | h-PARP14- sh3 | F:CCGGGCACCATTTGAAGAGTCACTACTCGAGTAGTGACTCT TCAAATGGTGCTTTTTG |
|  |  | R:AATTCAAAAAGCACCATTTGAAGAGTCACTACTCGAGTAGTGAC TCTTCAAATGGTGC |
| M-PARP14 | M-PARP14-sh1 | F:CCGGAGAATGTGACCAGATAGTAAATCGAG TTTACTATCTGGTCACATTCT TTTTTG |
|  |  | R: AATT CAAAAAAGAA TGTGACCAGA TAGTAA ACTC GAGTTTACTA TCTGGTCACA TTCT |
|  | M-PARP14-sh2 | F:CCGGAGCTCTTGTGCAACCATTATACTCGAG TATAATGGTTGCACAAGAGCTTTTTTG |
|  |  | R: AATT CAAAAAAGCT CTTGTGCAAC CATTAT ACTC GAGTATAATG GTTGCACAAG AGCT |
|  | M-PARP14-sh3 | F:CCGGCTACGGGAAAGGAACCTATTTTCGAGAAATAGGTTC CTTTCCCGTAGTTTTTG |
|  |  | R:AATTCAAAAACTACGGGAAAGGAACCTATTTCTCGAGAAATAGG TTCCTTTCCCGTAG |

**B. qPCR Primer Sequences.**

| Species | Gene | Forward (5’→3’) | Reverse (5’→3’) |
| --- | --- | --- | --- |
| Homo  sapiens | RPL11 | AAAGGTGCGGGAGTATGAGTT | TCCAGGCCGTAGATACCAATG |
|  | 18s | GTAACCCGTTGAACCCCATT | CCATCCAATCGGTAGTAGCG |
|  | 28s | CAGGGGAATCCGACTGTTTA | ATGACGAGGCATTTGGCTAC |
|  | EEF1A1 | TGTCGTCATTGGACACGTAGA | ACGCTCAGCTTTCAGTTTATCC |
|  | PARP14 | TGTTAGTGGAGAACATAAGTGGC | TGAATGGTGCTTGGTACAATCAT |
|  | Actin | CATGTACGTTGCTATCCAGGC | CTCCTTAATGTCACGCACGAT |
|  | GAPDH | AAGGTGAAGGTCGGAGTCAACGGATTTG | CTCGCTCCTGGAAGATGGTGATGGGATT |
| Mus  musculus | RPL11 | ATGGCGCAAGATCAAGGGG | GACTGTGCAGTGAACAGCAAT |
|  | EEF1A1 | ACACGTAGATTCCGGCAAGTC | GATGGTTCGCTTGTCGATTCC |
|  | PARP14 | AGCAGTGGATCAGAAAAGACAG | GTCAGCACCATCTCGGATACT |
|  | Actin | GGCTGTATTCCCCTCCATCG | CCAGTTGGTAACAATGCCATGT |
|  | GAPDH | ACTCTTCCACCTTCGATGC | CCGTATTCATTGTCATACCAGG |

**C. Primer for ChIP-PCR**

| Gene | Forward (5’→3’) | Reverse (5’→3’) |
| --- | --- | --- |
| 1-p-RPL11-Human | GCCTGCACTCCCAGCCTT TA | GCGCTGTCTTGGCTTTCTGT |
| 2-p-RPL11-Human | TGAGACCAGCCTGGGTAACAG | GCAAATGCAGCTCAGGCTTC |
| 3-p-RPL11-Human | GGCTCCCAAAGTGCTGGGATT | TGCTCTGCCTCTTGCCTTCC |
| 1-p-PARP14-Human | CAGAGCCAAGGTGACAGGAG | GGAGTCTGGGTTCCTGCTTC |
| 2-p-PARP14-Human | TGGGACTACAGGCTTGAGTACC | CCCTACCCCTCCTCAAACTATC |
| 3-p-PARP14-Human | GAAGTAGGCGGGATTACAGGTG | TCAGCAGGAATGGGAGAAAGTC |
| 4-p-PARP14-Human | AAGGGAAGGGAGAGGGAAAG | TTGGGTTGGGTTAGGGTTTG |
| Negative control | TACTAGCGGTTTTACGGGCG | TCGAACAGGAGGAGCAGAGAG C |

**D. Primary Antibodies Used in This Study.**

| Antibody | Application | Dilution | Company | Catalog No. |
| --- | --- | --- | --- | --- |
| H3K18la | WB, IHC, ChIP | 1:1000, 1:200 | PTM Biolabs | PTM-1406RM |
| Pan-Kla | WB | 1:1000 | PTM Biolabs | PTM-1401 |
| RPL11 | WB, IHC, IF | 1:2000, 1:500 | Proteintech | 16298-1-AP |
| EEF1A1 | WB, IF | 1:5000, 1:200 | Abcam | ab150311 |
| PARP14 | WB, IHC, IF | 1:1000, 1:200 | GeneTex | GTX116174 |
| γ-H2AX (pS139) | IF, WB | 1:400, 1:1000 | ABclonal | AP0099 |
| EP300 | WB, ChIP | 1:1000 | Cell Signaling Tech | 86377S |
| Ki67 | IHC | 1:200 | Abcam | ab16667 |
| GAPDH | WB | 1:10000 | Proteintech | 60004-1-Ig |
| Histone H3 | WB | 1:5000 | Cell Signaling Tech | 4499S |
| LDHA | WB | 1:5000 | Proteintech | 21799-1-AP |

**Supplementary Table S2. Pan-Kla IP Significant Differential Protein List**

**Supplementary Table S3. H3K18la CHIP_peakAnno**

**Supplementary Table S4. Rio-seq Functional Enrichment Analysis**

**Supplementary Table S5. Rio-seq ORF Differential Analysis**

**Supplementary Table S6. Analysis of public databases (GSE62452, GSE16515) reveals the correlation between RPL11 and EEF1A1 expression**

**Supplementary Table S7. RNA-sequencing (RNA-seq) on RPL11-knockdown PDAC resistant cells.** Ps：Due to their large size, Supplementary Tables S2-7 are provided as separate Excel files！
